# Modulatory role of RNA helicases in MBNL-dependent alternative splicing regulation

**DOI:** 10.1007/s00018-023-04927-0

**Published:** 2023-10-26

**Authors:** Katarzyna Taylor, Agnieszka Piasecka, Arkadiusz Kajdasz, Aleksandra Brzęk, Micaela Polay Espinoza, Cyril F. Bourgeois, Artur Jankowski, Małgorzata Borowiak, Katarzyna D. Raczyńska, Łukasz J. Sznajder, Krzysztof Sobczak

**Affiliations:** 1 Department of Gene Expression, Institute of Molecular Biology and Biotechnology, Adam Mickiewicz University, Uniwersytetu Poznanskiego 6, 61-614 Poznan, Poland; 2 Institute of Bioorganic Chemistry, Polish Academy of Sciences, Noskowskiego 12/14, 61-704 Poznan, Poland; 3 Laboratoire de Biologie et Modelisation de la Cellule, Ecole Normale Superieure de Lyon, CNRS, UMR 5239, Inserm, U1293, Universite Claude Bernard Lyon 1, 46 Allee d’Italie, 69364 Lyon, France; 4 Department of Molecular Genetics and Microbiology, Center for NeuroGenetics and the Genetics Institute, University of Florida, College of Medicine, Gainesville, FL 32610 USA; 5 Department of Chemistry and Biochemistry, University of Nevada, Las Vegas, NV 89154 USA

**Keywords:** RNA binding protein, RNA–protein interaction, RNA metabolism and maturation

## Abstract

Muscleblind-like splicing regulators (MBNLs) activate or repress the inclusion of alternative splicing (AS) events, enabling the developmental transition of fetal mRNA splicing isoforms to their adult forms. Herein, we sought to elaborate the mechanism by which MBNLs mediate AS related to biological processes. We evaluated the functional role of DEAD-box (DDX) RNA helicases, DDX5 and DDX17 in MBNL-dependent AS regulation. Whole-transcriptome analysis and validation approaches revealed a handful of MBNLs-dependent AS events to be affected by DDX5 and DDX17 in mostly an opposite manner. The opposite expression patterns of these two groups of factors during muscle development and coordination of fetal-to-adult splicing transition indicate the importance of these proteins at early stages of development. The identified pathways of how the helicases modulate MBNL splicing activity include DDX5 and DDX17-dependent changes in the ratio of MBNL splicing isoforms and most likely changes in accessibility of MBNL-binding sites. Another pathway involves the mode of action of the helicases independent of MBNL activity. These findings lead to a deeper understanding of the network of interdependencies between RNA-binding proteins and constitute a valuable element in the discussion on developmental homeostasis and pathological states in which the studied protein factors play a significant role.

**Supplementary Information:**

The online version contains supplementary material available at 10.1007/s00018-023-04927-0.

## Introduction

Alternative splicing (AS) is a complex process that contributes to profound diversity of the transcriptome and, consequently, homeostasis of the cellular proteome in response to various stimuli, including environmental, developmental and tissue-specific stimuli [1–3]. The outcome of AS is shaped by a network of diverse interactions between the main components of the splicing machinery, including small nuclear ribonucleoproteins, *cis*-acting splicing signatures including 5′ and 3′ splice sites (ss), a branch point and polypyrimidine tract as well as other RNA regulatory elements and RNA-binding proteins (RBPs) [4]. The functionality of these RNA elements and the activity of RBPs reflected in the AS profile are defined by a subset of key determinants under spatiotemporal pressure, including RNA structure, which affects the accessibility of functional *cis*-acting regulatory elements [5], RBP expression levels [6] and their isoform ratio [7, 8].

Muscleblind-like splicing regulators (MBNLs) as an alternative splicing factor play an essential role in fetal-to-adult splicing transition. MBNL1 and MBNL2 (MBNL1,2) are the major paralogs of mostly redundant functions and their simultaneous knock down leads to an increased effect on splicing changes of the same target RNAs [9–12]. Moreover, a knock down of either paralog induces increased production of the other one [12, 13]. Nevertheless, their biological expression differs with MBNL1 playing a major function in muscular and immune system development where its expression prevails [14–18]. Increasing expression of MBNL1 has been found during myoblast differentiation [19, 20] and skeletal muscle and heart development [15, 21]. MBNL2 is prominently expressed in the brain and is crucial in neurogenesis [13, 22]. All MBNL paralogs can regulate the inclusion or exclusion of the same AS events with different activities [9]. Additionally, each paralog is represented by a subset of splicing isoforms that differ in the presence of regions encoded by alternative exons (e; e1, e3, e5, e7, e8) [23]. These exons convey distinct properties including intracellular localization [24], RNA-RNA binding efficiency [25], protein–protein interaction [26] or homo-typic interactions [7, 27]. Different splicing activity of the paralogs and isoforms seems to be important during developmental processes as well as in pathological states such as tumorigenesis and multisystem diseases, including myotonic dystrophy types 1 and 2 (DM1 and DM2) [7–9, 28].

Multiple research groups have mutually proposed the specific recognition of an RNA motif consisting of a GpC dinucleotide flanked by pyrimidines, YGCY (Y–U or C), by all three MBNL paralogs [9, 29]. The numbers of these motifs close to one another, the secondary structures they are encompassed in and their locations within pre-mRNAs constitute significant determinants of MBNL splicing activity [30, 31]. Interaction of MBNLs with multiple YGCY motifs within an alternative exon or up to 300 nucleotides upstream induces skipping of the exon (e_OFF). This may occur when the association of U2 auxiliary factor 65 kDa subunit (U2AF65) with the 3’ss is impeded through RNA structural changes or steric hindrance, as was shown for *TNNT2* e5 [32]. On the other hand, binding of MBNLs within 500 nucleotides of a downstream intron promotes the inclusion of an alternative exon (e_ON) into mRNAs [29], potentially due to an effect on spliceosome formation by the enhancement of U2AF65 binding, as was proposed in studies on the inclusion of *INSR* e7 [33].

The mechanism by which MBNLs mediate the inclusion or skipping of AS events is strongly expected to be complex and still little is known in this matter. The regulation of AS events has been described between MBNL and other RBPs including DDX helicases [34]. Interestingly, MBNL splicing activity relies on the secondary structure of RNA *cis*-acting regulatory elements [30, 31], whereas RNA helicases modulate the structural organization of pre-mRNA. It is unclear, however, to what extent the helicases impact MBNL-dependent splicing and whether it has a biological relevance. Until now, two paralogs, DDX5 and DDX17 (DDX5,17) have been shown to coimmunoprecipitate with MBNL1 under weak ionic conditions [35], and DDX5 was found to facilitate MBNL1-induced exclusion of *TNNT2* e5 by structurally changing the availability of MBNL-binding sites [34]. Similar to MBNLs, DDX5,17 have been described as important players in the regulation of AS [36–39] and other molecular pathways that direct developmental processes, including adipogenesis [40], myoblast differentiation [37, 41], neurogenesis [42] and pluripotency determination [43].

DDX5,17 regulate gene expression at multiple levels [44] and due to cross-regulation of both proteins’ expression [45] they are mainly analyzed inseparably [37, 39, 45–48]. Their functional contribution to AS has been crucial [49, 50] and includes determination of mRNA splicing isoforms of DNA-binding proteins and RBPs [37, 39]. To date, two functions of DDX helicases in the regulation of AS have been considered. First, they can rearrange GC-rich RNA structures through mostly unwinding inter- or intramolecular interactions [36, 37, 51] or even anneal single-stranded regions [52]. This may either facilitate the binding of splicing factors, including U1 snRNP, hnRNP F or MBNL1 itself, to *cis*-acting RNA regulatory elements that are otherwise inaccessible [34, 36, 37, 53] or block their binding, as was described for hnRNP H [54]. The function of RNA helicases may also involve the remodeling of protein-RNA complexes by directly disrupting or facilitating interactions between RNA and protein components through protein–protein interaction of the helicases with encountered RBPs in an RNA-bound or unbound state [50, 51, 55–57].

Herein, we tested a hypothesis that DDX5,17 and MBNL1,2 coregulate many studied AS events during developmental processes. We uncovered a modulatory role of DDX5,17 in regulation of a handful of MBNL1,2 dependent AS events and its relevance in skeletal muscle development. We identified DDX5,17 and MBNL1,2 as predominantly antagonistic AS regulators. While MBNL1,2 mostly induce the fetal-to-adult AS transition, DDX5,17 promote the splicing in both directions equally  in analyzed transcripts. We also discovered that Ddx5 and Mbnl1 expression follows opposite patterns during murine muscle development. Finally, we identified three molecular pathways underlying antagonistic nature of AS regulation by DDX5,17 and MBNL1,2, including functional modulation of both the ratio of *MBNL1* isoforms that differ in splicing activities and most likely accessibility of MBNL-binding sites. DDX5,17 also regulate shared AS events, most likely in cooperation with additional RBPs that antagonize or work alongside MBNLs.

## Materials and methods

### Genetic construct preparation

The *MBNL1* e5 minigene, analogous to what was previously described in [58], was prepared as follows. A region containing 51 nucleotides from the 3′-end of intron 3, exon 4, intron 4, exon 5, intron 5, exon 6, and 33 nucleotides of the 5′-end of intron 6 was amplified from HeLa genomic DNA using PCR primers carrying a 15-nt overhang complementary to the pcDNA3.1(−) plasmid. The PCR fragment was inserted into linearized pcDNA3.1(−) between the NotI and BamHI restriction sites. MBNL1 fragment amplification and linearization of pcDNA3.1(−) were carried out using Phusion HiFi DNA polymerase (Thermo Fisher) and CloneAmp HiFi PCR premix (Takara Bio), respectively. An In-Fusion HD Cloning kit (Takara Bio) was used for fast and directional cloning. The primer sets are listed in Suppl. Table S1. The pEGFP-MBNL-41 expression vector and *Atp2a1* e22 splicing minigenes (Atp2a1-BS and Atp2a1-ΔBS) have been previously described [31, 59]. The *Nfix* e7 splicing minigenes (Nfix-BS and Nfix-mtBS) were a gift from Manuel Ares Jr. [60].

### Cell culture and transfection

WT and Mbnl1,2 DKO MEFs were gifts from Maurice Swanson. HeLa cells (ATCC), MCF7 cells and WT and Mbnl1,2 DKO MEFs were grown in high-glucose DMEM (Lonza) supplemented with 10% fetal bovine serum (FBS) (Sigma Aldrich) and 1 × penicillin–streptomycin (Sigma Aldrich). HSkM (Life Technologies) cells were grown in HAM F-10 medium (Sigma Aldrich) supplemented with 20% FBS, 1 × penicillin–streptomycin, 0.39 µg/mL dexamethasone (Sigma Aldrich) and 10 ng/mL epidermal growth (Sigma Aldrich). All cells were grown at 37 °C and an atmosphere containing 5% CO_2_. Prior to transfection, the cells were plated in 6-well or 12-well plates and transfected at 50–60% confluence with plasmids using Lipofectamine 3000 (Thermo Fisher) or with siRNA or antisense oligonucleotides using Lipofectamine RNAiMAX (Thermo Fisher) according to the manufacturer’s protocol. Genes were knocked down with siRNAs against *MBNL1*, *MBNL2*, *DDX5*, *DDX17* or control siRNA was used (siCtrl) [9, 37] (Sigma Aldrich) at 50 nM. SSOs with 2′-*O*-methoxyethyl-phosphorothioate (2′MOE-PS) modification, SSO-e7 and SSO-Ctrl, were used at 25 nM. Cotransfection with 200 ng of the *ATP2A1* e22 minigenes and 250 ng of pEGFP-MBNL1-41 expression vector was preceded by siRNA treatment and  4 h of incubation period. The cells were harvested 48 h after transfection. The siRNA and SSO sequences are listed in Suppl. Table S1.

### Preparation of stable shDDX5, shDDX17 and shCtrl HeLa cell lines

A lentiviral vector for doxycycline-inducible DDX5,17 KD was constructed by inserting annealed and phosphorylated oligonucleotides (GGCTAGATGTGGAAGATGT) into the AgeI/EcoRI sites of the pLKO-Tet-On plasmid (a gift from Dmitri Wiederschain; Addgene plasmid # 21,915 as described in [61] to create pLKO-Tet-On-shDDX5/17. The shRNA is expressed from this vector under the H1 promoter and is further converted into siRNA that targets nucleotides 1381–1399 of DDX5 mRNA (numbering according to ENST00000225792) and nucleotides 1516–1534 of DDX17 mRNA (numbering according to ENST00000403230.3). A control lentiviral vector with an inducible shRNA scramble expression cassette was ordered from Addgene (a gift from David Sabatini; Addgene plasmid # 1864 as described in [61]). Virus production and HeLa cells transduction with DDX5,17 shRNA and scramble shRNA were performed as follows: HEK 293 T cells were transfected with pLKO-Tet-OnshDDX5,17 and scramble shRNA supplemented with packaging and envelope vector, psPAX2 and pMD2.G, respectively, by the calcium phosphate method as described previously [61]. Fresh medium was added to the cells 24 h after transfection, and lentiviral supernatants were collected 72 h after transfection. For transduction, HeLa cells were incubated with lentiviral supernatants supplemented with 4 μg/mL polybrene (hexadimethrine bromide, Sigma Aldrich) for 14 h, and fresh medium was then added. HeLa cells transfected with DDX5,17 shRNA or scramble shRNA were selected by adding puromycin to a final concentration of 0.3 μg/mL for 7 days. For siRNA-induced silencing, cells were treated with 100 ng/ml of doxycycline for 5 days.

### Mouse tissue collection and RNA/protein extraction

This study received ethical approval from the National Ethics Committee for Animal Testing. All animal procedures and endpoints were in accordance with the ARRIVE guidelines and animals were sacrificed in accordance with the National Ethics Committee for Animal Testing and Polish guidelines and regulations. Mice were housed under specific pathogen free conditions in the animal facility of the Center of Advanced Technologies, Adam Mickiewicz University, Poznan, Poland. Three adult CDC-1 IGS female mice were sacrificed at day 365 for brain, skeletal muscles and heart collection. Two CDC-1 IGS mice were sacrificed at day 19 of fetal development, day 1 and 5 of postnatal development for quadriceps collection (E19, PN1, PN5, respectively). Protein extraction was carried out in RIPA buffer (150 mM NaCl, 50 mM Tris–HCl (pH 8.0), 1 mM EDTA, 0.5% NP-40, 0.5% Triton X-100, 0.5% sodium deoxycholate, 0.1% SDS) supplemented with EDTA-free Halt™ Protease and Phosphatase Inhibitor Cocktail (100X), using a bead-based homogenizer (TissueLyser II, Qiagen) (2 × 45 s, max frequency) and 1.4-mm and 2.8-mm ceramic beads (Qiagen). The homogenized lysate was incubated on ice for 30 min with vortexing every 10 min and centrifuged at 18 000×*g* at 4 °C for 15 min. The collection of mouse samples from 5 stages of skeletal muscle, heart and brain development followed by RNA isolation is described elsewhere [62].

### Splicing and expression analyses of precursor and mature mRNA

HeLa, HSkM and MEFs were prepared in TRI reagent (Sigma Aldrich), followed by rapid RNA purification using a Total RNA Zol-out kit (A&A Biotechnology) including DNase treatment according to the manufacturer’s protocol. Total RNA from healthy adult human skeletal muscles derived from male individuals at ages 24, 43 and 76 was purchased (BioChain). Total RNA (1–2 µg) was reverse transcribed using random hexamers and a TranScriba Kit (A&A Biotechnology) according to the manufacturer’s protocol. Splicing analyses of the MBNL1 exon 5 minigene were conducted with a forward. All PCR experiments were conducted with GoTaq Flexi DNA polymerase (Promega). PCR products were resolved on agarose gels with ethidium bromide (Sigma Aldrich) and visualized on G:BOX (Syngene), followed by signal analyses using Multi Gauge v3.0 software. Splicing calculation referred to a percent spliced-in (PSI) value and ΔPSI, which represents a difference in PSI between the control and tested conditions. Primers designed or adopted from other articles [9, 31, 63] were amplified predominantly across a single splicing event. The majority of the tested splicing events were relatively short (50–200 nt long), which helped to reduce amplification bias between splice isoforms with alternative exon inclusion or exclusion. Quantitative PCR (qPCR) was conducted with Maxima SYBR Green Rox (Thermo Fisher) on a QuantStudio 7 Flex instrument (Thermo Fisher). Primer sequences are provided in Suppl. Table S1.

### Immunoblotting

HeLa, HSkM or MEF cells were lysed with RIPA buffer supplemented with EDTA-free Halt™ Protease and Phosphatase Inhibitor Cocktail (100X). Lysates were incubated on ice for 30 min, frozen at − 80 °C O/N and centrifuged at 18 000×*g* at 4 °C for 15 min. Protein concentrations were detected using a Pierce BCA Protein Assay Kit (Thermo Fisher). Samples (60 μg for DDX5 detection in HSkM or 20–30 µg for all other proteins) were heated to 95 °C for 5 min in SDS-loading buffer and separated on 8 or 10% SDS polyacrylamide gels or prepared for and separated on gradient Bolt™ 4–12% Bis–Tris Plus Gels (Thermo Fisher) according to the manufacturer’s protocol, followed by transfer to 0.45-µm PVDF membranes (Sigma Aldrich) using a wet transfer apparatus (1 h, 100 V, 4 °C). Membranes were blocked for 1 h with 5% skim milk in PBST buffer (phosphate-buffered saline (PBS) with 0.1% Tween-20; for MBNL1, MBNL2, DDX5, GAPDH) or TBST buffer (Tris buffered saline with 0.1% Tween-20; for VINCULIN and DDX17) and incubated with primary antibodies against MBNL1 (A2654; gift from Thornton), MBNL2 (sc-136–167, Santa Cruz), DDX5 (D15E10; Cell Signaling), DDX17 (NB200-352; Novusbio), human GAPDH (sc-47724; Santa Cruz) or human/mouse VINCULIN (EPR8185, Abcam). Anti-rabbit (A9169, Sigma Aldrich) and anti-mouse (12–349, Millipore) secondary antibodies were conjugated with horseradish peroxidase, detected using Immobilon Forte Western HRP Substrate (Sigma Aldrich) and visualized on G:BOX (Syngene).

### RNA immunoprecipitation

RIP was performed using MCF7 cells with or without an induced expression of DDX-HA wildtype or DDX5-HA mutant (K144A). After 48 h treatment with 0.5 μg/mL doxycycline a whole cell extract was prepared (WCE)the cells were collected and lysed in RIPA buffer (150 mM NaCl, 50 mM Tris–HCl (pH 8.0), 1 mM EDTA, 0.5% NP-40, 0.5% Triton X-100, 0.5% sodium deoxycholate, 0.1% SDS) supplemented with EDTA-free Halt™ protease inhibitor, 1 mM DTT, 40U RNasin^®^ (Promega). Lysates were incubated on ice for 30 min and then incubated at − 80 °C O/N. Dynabeads Protein A (Invitrogen) were incubated with 7.5 μg HA-tag antibody (Abcam, 9110) on rotating wheel O/N at 4 °C. WCE was thawed and homogenized by passing the lysate 5 times through 25-gauge needle and centrifuged at 2000 rpm for 10 min at 4 °C. 15% of the WCE was collected for INPUT. The WCE was incubated with the beads on rotating wheel for 2 h at 4 °C followed by three rounds of washing with PBST (137 mM NaCl, 2.7 mM KCl, 8 mM Na_2_HPO_4_, and 2 mM KH_2_PO_4_, 0.02% Tween-20) and elution with acidic glycine solution. RNA was isolated using PureLink RNA mini kit (Thermo Fisher). RIP qPCR results were represented as relative IP/INPUT ratio = 2^(Ct(IP)^ − Ct(INPUT)) − (Ct(IP ctrl) − Ct(INPUT-ctrl)).

### Quantification of the protein-RNA interaction in vitro

The electrophoretic mobility shift assay (EMSA) was carried out by incubation of 15 nM of 5′-biotynylated and 3′-Cyanine 5 labelled RNAs (Sigma Aldrich) with the indicated concentrations of recombinant rMBNL1 (H00004154-P02, Abnova) and rDDX5 (TP300371, OriGene). The reactions were performed in a volume of 10 µl in buffer A (50 mM NaCl, 50 mM KCl, 50 mM Tris–HCl, 1 mM MgCl_2_) at 37 °C for 15 min. The samples were mixed with loading buffer (30% glycerol) and run on a non-denaturing 6% PA gel. Gels were scanned using Amersham Typhoon RGB Biomolecular Imager. Cyanine 5 fluorescence was excited by 635 nm and detected using a Cy5 filter. RNA-DDX5 interaction was calculated based on the signal of free RNA which was extrapolated into RNA–protein complexes. A dissociation constant, *K*_d_, was calculated using the equation: one site specific binding *Y* = *B*_max_*X/(*K*_d_ + *X*) with *B*_max_ = 100, in Graph Pad program.

### RNA pull down assay

The RNA pull down assay was carried out using Dynabeads™ MyOne™ Streptavidin C1 (65,001, Thermo-Fisher) blocked with 2% UltraPure™ BSA (Ambion). The following reagents: 2 nmol of 5′-biotynylated and 3′-Cyanine 5 labelled RNA (Sigma Aldrich), 100 nM rMBNL1 (H00004154-P02, Abnova) and 400 nM rDDX5 (TP300371, OriGene) or 400 nM BSA were incubated in PBST buffer (137 mM NaCl, 2.7 mM KCl, 8 mM Na_2_HPO_4_, and 2 mM KH_2_PO_4_, 0.02% Tween-20, pH 8.0) with 10U RNasin^®^ (Promega) in a total volume of 20 μL for 15 min at 37 °C. The reaction was combined with the beads and incubated for 15 min at RT followed by a collection of fraction #2 of unbound proteins and three rounds of washing steps with PBST. The beads were transferred to a new tube and elution of RNA–protein complexes (fraction #1) was performed by adding 4 × Bolt™ LDS sample buffer and 10 × reducing agent (Thermo Fisher). All samples were heated to 95 °C for 5 min and separated on a gradient Bolt™ 4–12% Bis–Tris Plus gel according to the manufacturer’s protocol. Immunoblotting was performed as above mentioned for MBNL1 antibody.

### RNA-seq data analyses

Publicly available RNA-seq data were retrieved from public repositories including DDX5,17 KD HEK293T cells (GSE123752; [46]), MBNL1,2 KD HeLa cells (PRJNA195384; [10]), MBNL1 KD HFE-145 cells (PRJEB32567; [64]) and DDX5 KD K562 cells (GSE11544; [38]), development of skeletal muscles (GSE108402; [16]) and the brain (SRP055008; [65]). Reads were aligned to the human hg38 or mouse mm10 genomes. Salmon [66] was used for transcript expression quantification and differential gene expression analysis was performed using DESeq2 [67]. For AS analysis, reads were aligned using STAR [68] followed by rMATS [69] (version 4) analysis. Results of AS analyses and expression analyses and are listed in Suppl. Table S2.

### DDX5 RIPseq data analyses

Raw reads from GSE175455 were mapped to the human genome (Ensembl GRCh38) with STAR (v2.7.10b) [68]. To build a transcriptome index, which was used during the aligment with STAR, gene annotations from Ensembl release 104 were employed. Enrichment of coverage by DDX5 was identified using MACS (v3.0.0a6) [70] by comparing DDX5 IP to input control with *Q* value set to 0.05. To visualize results bedGraphs files, generated by MACS, were uploaded into UCSC genome browser. The significant DDX5 peaks were assigned to the genes with R GenomicRanges and GenomicFeatures packages [71].

### Statistical analyses

Statistical analyses were performed using the GraphPad program for at least 3 biological replicates (*n*) or as specified in the figures. Linear association between variables and its statistical significance was determined by Pearson’s correlation coefficient test. Statistical significance for RT-PCR splicing analyses and protein levels is expressed as the mean ± standard deviation (SD) and was determined by the two-tailed Student’s *t* test (ns, nonsignificant, ∗ for *P* < 0.05, ∗∗ for *P* < 0.01, ∗∗∗ for *P* < 0.001).

## Results

### DDX5,17 and MBNL1,2 promote opposite splicing patterns of MBNL-dependent exons

**Fig. 1:**
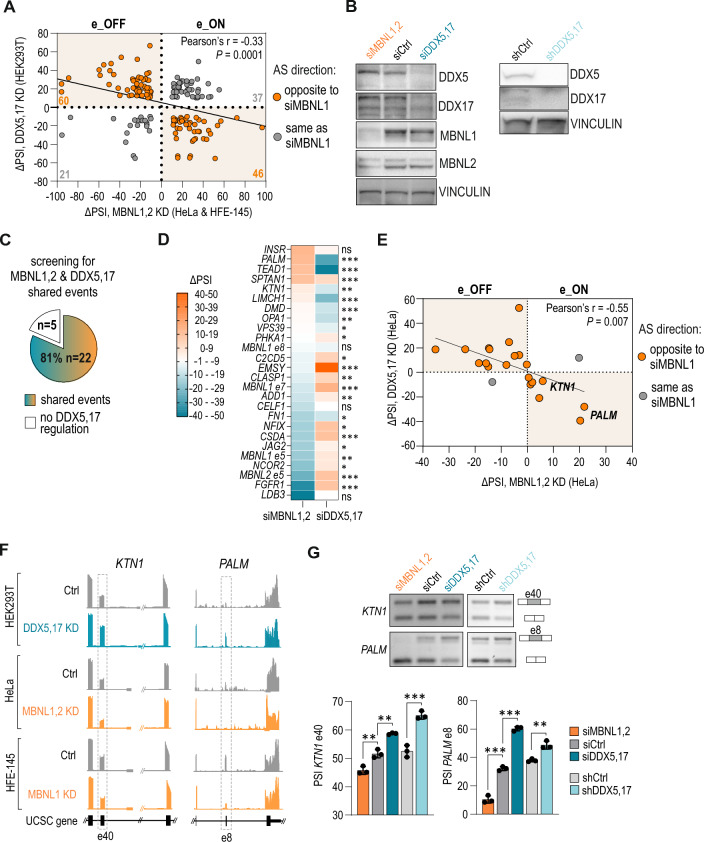
DDX5,17 are splicing modulators of a large subset of MBNL-dependent AS events. **A** AS events identified in RNA-seq AS analyses and shared by MBNL1,2 and DDX5,17 show negatively correlated regulation. Computed ΔPSI values from RNA-seq AS analyses were compared between DDX5,17 KD HEK293T cells (*n* = 3; [46]), MBNL1,2 KD HeLa cells (*n* = 1; [10]) and MBNL1 KD HFE-145 cells (*n* = 3; [64]). Exons excluded (e_OFF) and included (e_ON) by MBNLs. The strength of a linear association and its statistical significance was determined by Pearson’s correlation coefficient. **B** Representative western blot analyses of protein levels in HeLa cells upon siRNA treatment or shRNA expression (*n* = 3). VINCULIN serves as a loading control. Blot images of all samples and calculated protein levels are shown in Suppl. Figure S1A. **C** The pie chart summarizes the RT-PCR screening for events shared by MBNL1,2 and DDX5,17 in siDDX5,17- and siMBNL1,2-treated HeLa cells among 27 selected MBNL-dependent AS events. The AS events were categorized into a group of shared events if PSI ≥ 5% and *P* < 0.05 in Student’s *t* test. AS analyses of each event are described in Suppl. Figure S1. **D** The heatmap presents AS events shown in C which changed or were insensitive to DDX5,17 depletion compared to MBNL1,2 KD conditions tested in HeLa cells by RT-PCR (*n* = 3). Significant difference was determined by Student’s *t* test; *ns* nonsignificant, ∗ for *P* < 0.05, ∗∗ for *P* < 0.01, ∗∗∗ for *P* < 0.001. **E** Negative correlation between ΔPSI values detected by RT-PCR for 27 shared AS events in DDX5,17 KD and MBNL1,2 KD HeLa cells compared to control conditions. The strength of a linear association and its statistical significance was determined by Pearson’s correlation coefficient. **F** Genome browser view (hg38) of RNA-seq AS analyses presented in **A** for *KTN1* e40 and *PALM* e8 events with surrounding exons. **G** Representative gels of RT-PCR validation and calculations show that DDX5,17 depletion promoted exon inclusion of *KTN1* e40 and *PALM* e8. Data represent the mean PSI values ± SD (*n* = 3). Statistical significance was calculated in reference to control (siCtrl) using Student’s *t* test; ∗∗ for *P* < 0.01, ∗∗∗ for *P* < 0.001

To test the hypothesis that DDX5,17 have a significant impact on MBNL1,2-dependent regulation of AS, we aimed to identify high-confidence AS events shared by DDX5,17 and MBNL1,2 in the human transcriptome. First, we retrieved publicly available RNA sequencing (RNA-seq) datasets from MBNL1 knock-down (KD) HFE-145 cells [64], MBNL1,2 KD HeLa cells [10] and DDX5,17 KD HEK293T cells [46]. We focused on changes in splicing of simple cassette events measured as the difference in the rate of exon inclusion (percentage spliced in, PSI) exceeding or equal to 10% (|ΔPSI|≥ 10; FDR ≤ 0.05). AS analyses revealed 2,225 unique MBNL1,2-dependent AS events in both MBNL KD models and 2434 DDX5,17-regulated AS events (Suppl. Figure S1A, Suppl. Table S2). We identified 164 shared events regulated by both classes of RBPs, which constituted 7.4% of the detected MBNL1,2-dependent AS events and consisted of both exon inclusion (e_ON) and exclusion (e_OFF) events (Fig. 1A, Suppl. Table S2). Interestingly, we noticed that 65% of these shared AS events were regulated in opposite ways in DDX5,17- and MBNL1,2-depleted cells (Fig. 1A; orange dots). It is, however, worth mentioning that conducted analyses were performed on datasets obtained by different experimental approaches and that they derive from essentially different cellular models. Even though these results do not provide explicit conclusions, they do indicate some degree dependency of studied splicing factors which we further elaborate in more detail.

Next, we selected 27 AS events out of 63 analyzed ones, with detectable two splicing isoforms which were previously shown to be MBNL-dependent either through RT-PCR or in RNA-seq or emerged in this study (Suppl. Table S1). We tested their sensitivity to DDX5,17 depletion and the direction of splicing changes. We chose HeLa cells as a model for further studies based on their considerable expression level of MBNL1,2 and DDX5,17. Since MBNL1 and MBNL2 as well as DDX5 and DDX17, often display redundant functions in AS regulation [9, 37, 39, 48], we performed simultaneous MBNL1,2 KD or DDX5,17 KD using siRNAs (Fig. 1B, Suppl. Figure S1B). We also established a stable HeLa cell line with a doxycycline-induced expression of shRNA targeting both helicases (Fig. 1B). RT-PCR analyses of all tested MBNL1,2-dependent AS events in siRNA-treated cells revealed that the great majority (81%) were sensitive to DDX5,17 depletion (Fig. 1C, Suppl. Figure S1C). Consistent results were obtained in the shRNA-inducible HeLa cell model (Suppl. Figure S1C). Interestingly, the splicing direction of as much as 90% of these shared events in DDX5,17 KD cells was opposite to that in MBNL1,2 KD cells (Fig. 1D). Exon inclusions caused by DDX5,17 KD and that caused by MBNL1,2 KD were negatively correlated (Fig. 1E). For example, MBNL1,2 KD and DDX5,17 KD had an opposite effect on *KTN1* e40 and *PALM* e8 inclusion in multiple models, as shown by RNA-seq AS analyses and RT-PCR (Fig. 1F, G). Furthermore, the reconstitution of DDX5 partially restored splicing of all selected shared events while a DDX5 mutant lacking RNA helicase activity had no effect on splicing of most of them (Suppl. Figure S1D, E). Collectively, our results show that DDX5,17 promote opposite splicing pattern to MBNLs for most studied MBNL-dependent AS events.

### DDX5,17 regulate MBNL-dependent developmental splicing pattern of studied events

**Fig. 2:**
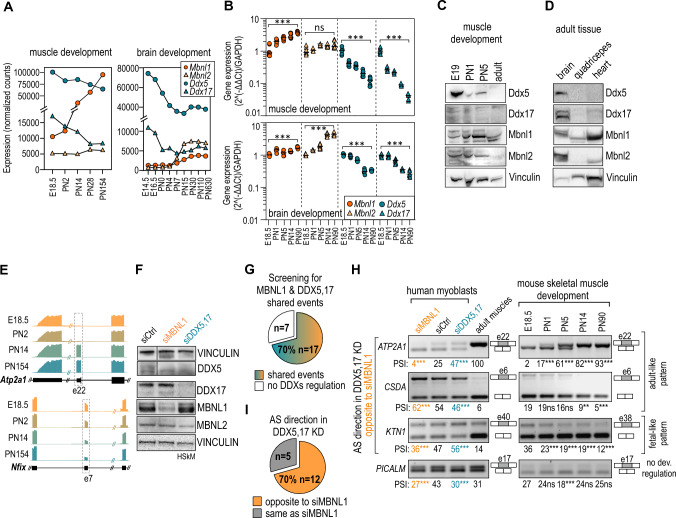
DDX5,17 affect the splicing of developmentally-regulated and MBNL-dependent AS events in muscle cells. **A** *Mbnl1*, *Mbnl2*, *Ddx5* and *Ddx17* gene expression during development of skeletal muscles (*n* = 1; [16]) and the brain (*n* = 2; [65]) determined by RNA-Seq expression analyses and using DESeq2-normalization method. E and PN correspond to embryonic and postnatal stages of development, respectively. **B** *Mbnl1*, *Mbnl2*, *Ddx5* and *Ddx17* gene expression across five stages of murine muscle development (E18.5, PN1, PN5, PN14, PN90) determined by RT-qPCR. Data represent the mean ± SD (*n* = 9). Statistical significance was calculated in reference to embryonic day 18.5 (E18.5) using Student’s *t* test; *ns* nonsignificant, ∗∗∗ for *P* < 0.001. **C** Representative western blot analyses of Mbnl1, Mbnl2, Ddx5 and Ddx17 protein levels in four murine muscle developmental stages (E19, PN1, PN5, adult ) (*n* = 2). Vinculin serves as a loading control. **D** Representative western blot analyses of Mbnl1, Mbnl2, Ddx5 and Ddx17 protein levels in adult murine skeletal muscles, heart and brain (*n* = 3). Vinculin serves as a loading control. **E** Genome browser view (mm10) of RNA-seq AS analyses shows MBNL-dependent AS events, *Atp2a1* e22 and *Nfix* e7, whose splicing changes during muscle development. **F** Representative western blot analyses of protein levels in HSkM upon siDDX5,17 treatment. VINCULIN serves as loading controls. Each loading control corresponds to the proteins above it. The image presents juxtaposed lanes that were non-adjacent in the blot. The lanes separation is delineated by a black separation. Blot images of all samples and calculated protein levels are shown in Suppl. Figure S2C. All samples derive from the same experiment and the blots were processed in parallel. **G** The pie chart summarizes the RT-PCR screening for AS events shared by MBNL1 and DDX5,17 in siDDX5,17- and siMBNL1-treated HSkM. The AS events were categorized into a group of shared events if PSI ≥ 5% and *P* < 0.05 in Student’s *t* test. AS analyses of each event are presented in Suppl. Figure S2B. **H** Representative gels and calculations of RT-PCR analyses of shared AS events in siRNA treated HSkM (*n* = 3), human adult muscles (*n* = 3) and across five stages of murine muscle development (*n* = 4). Data represent the mean PSI values ± SD. Statistical significance was calculated in reference to control (siCtrl) or E18.5 using Student’s *t* test; *ns* nonsignificant, ∗ for *P* < 0.05, ∗∗ for *P* < 0.01, ∗∗∗ for *P* < 0.001. **I** The pie chart shows the number of shared AS events analyzed in HSkM, whose direction of splicing was opposite in DDX5,17 KD cells to that in MBNL1 KD cells (orange) or the same (gray). The AS events were categorized into these two groups if PSI ≥ 5% and *P* < 0.05 in Student’s *t* test

Previous studies demonstrated the importance of DDX5,17 in developmental processes including myogenesis and neurogenesis [37, 41, 42], in which MBNL1,2 also play an essential role by governing developmental splicing transition [14, 16]. To assess *DDX5,17* and *MBNL1,2* gene expression during tissue maturation, we retrieved publicly available RNA-seq datasets from developing mouse skeletal muscles [16] and the brain [65]. Our analysis showed a gradual decrease in *Ddx5* and *Ddx17* expression during development in both types of tissues (Fig. 2A, Suppl. Figure S2A). In contrast, the expression level of *Mbnl*s increased in a time course of development or remained the same in case of *Mbnl2* in muscle tissues. These results were further supported by the RT-qPCR analyses (Fig. 2B). To find out whether a change in RNA level of the helicases and *Mbnl*s is reflected in the protein production we collected and examined murine muscles in four stages of development, E19, PN1, PN5 and adult. The opposite expression pattern was mostly seen for Mbnl1 and Ddx5 (Fig. 2C, Suppl. Figure S2B). Interestingly, in adult skeletal muscles and heart we noticed a vast disproportion between the level of Mbnl1,2 and Ddx5,17 compared to the brain (Fig. 2D, Suppl. Figure S2C). Observed negative correlation between Ddx5 and Mbnl1 expression during development of skeletal muscles may suggest a potential modulatory role of the helicases in Mbnl-dependent splicing regulation during early stages of muscle development.

To test the hypothesis that DDX5,17 coregulate MBNL-dependent developmental splicing pattern, we asked how many shared AS events change during muscle development. For example, *Atp2a1* e22 and *Nfix* e7, two out of many MBNL-dependent events, undergo robust developmental transition (Fig. 2E). We performed splicing analysis of human myoblasts (HSkM) and adult skeletal muscles, and mouse muscles at different stages of development. Next, we selected 24 AS events out of 63 analyzed, with detectable two isoforms in HSkM cells which were previously shown to be MBNL-dependent either through RT-PCR or RNA-seq or emerged in this study (Suppl. Table S1). RT-PCR screening in DDX5,17 KD and MBNL1 KD HSkM revealed that 17 (70%) of these AS events were modulated by depletion of both classes of RBPs (Fig. 2F, G, Suppl. Figure S2D, E). As anticipated, the majority of the identified shared events (75%) undergo conserved splicing transition during muscle development (Fig. 2H, Suppl. Figure S2F). Their splicing patterns vastly differ in human adult muscles and control myoblasts and gradually change from embryonic to adult splicing patterns during mouse muscle development. In agreement with our observations from previous experiments in HeLa cells, most shared AS events (12 out of 17; 70%) showed opposite splicing patterns upon MBNL1 KD and DDX5,17 KD (Fig. 2 I, Suppl. Figure S2E). Among developmentally regulated AS events, DDX5,17 depletion equally promoted the expression of adult-like and fetal-like splicing isoforms (Fig. 2H, Suppl. Figure S2F). The significant contribution of Ddx5,17 to the Mbnl-regulated developmental splicing transition was also confirmed in a murine myogenesis model (Suppl. Figure S2G, H).

Taken together, the collected evidence shows the negative correlation of expression of both classes of RBPs during development and a significant effect of DDX5,17 loss on the AS pattern of studied MBNL-dependent events.

### DDX5 and DDX17 affect MBNL1 activity by changing MBNL1 splicing isoforms

We hypothesize that the antagonistic activity of DDX5,17 and MBNL1,2 in studied cellular models may involve several pathways: (I) a direct effect of DDX5,17 on MBNL1,2 expression and/or the splicing isoform ratio, (II) impairment of the functional interaction of MBNL1,2 with *cis*-acting RNA regulatory elements, and (III) independent regulation of shared AS event by DDX5,17 and MBNL1,2.

To test the first hypothesis, we analyzed publicly available RNA-seq data from two different cell lines with either DDX5 KD alone or combined DDX5,17 KD [38, 46]. We did not observe changes in expression levels of *MBNL1* or *MBNL2* (Suppl. Table S2). The total MBNL1 and MBNL2 protein levels were also unchanged upon DDX5,17 depletion in the studied by us HeLa and HSkM models (Figs. 1B, 2F, Suppl. Figure S3A). Since the MBNL1,2 proteins regulate several AS events in MBNL1,2 pre-mRNA, we asked whether DDX5,17 affect the ratio of MBNL splicing isoforms.

**Fig. 3:**
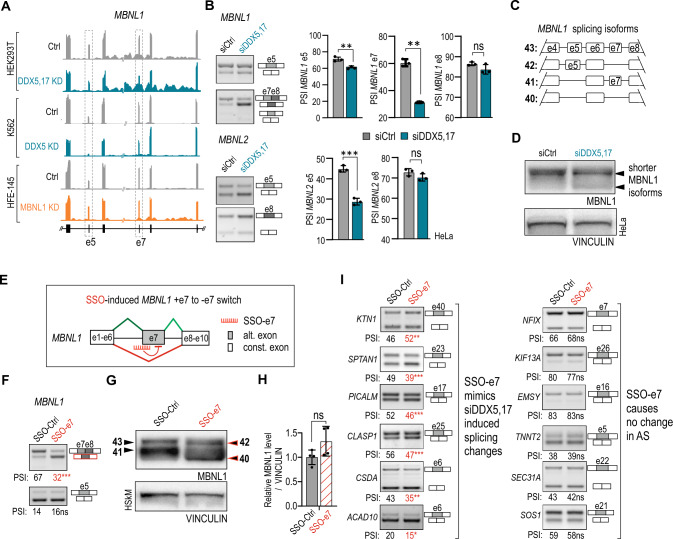
DDX5,17 affect MBNL1 activity by modulating the ratio of MBNL1 splicing isoforms. **A** Genome browser view (hg38) of RNA-seq AS analyses showing the AS pattern of *MBNL1* e5 and e7 in DDX5,17 KD HEK293T cells (*n* = 3; [46]), DDX5 KD K562 cells (*n* = 3; [38]) and MBNL1 KD HFE-145 cells (*n* = 3; [64]. **B** Representative gels and calculations of RT-PCR analyses of *MBNL1* and *MBNL2* AS events in siDDX5,17-treated HeLa cells. Data represent the mean ± SD (*n* = 3). Statistical significance was calculated in reference to siCtrl using Student’s *t* test; *ns* nonsignificant, ∗∗ for *P* < 0.01, ∗∗∗ for *P* < 0.001. **C** Graphical representation of distinct *MBNL1* alternative splicing isoform pre-mRNAs differing in the presence of AS events and considered in this study. Numerical naming of each *MBNL1* isoform: 40, 41, 42, 43, correspond to a molecular mass of the proteins in kDa and followes [23]. **D** Representative western blot analyses show different distributions of MBNL1 protein isoforms between control conditions and DDX5,17 KD HeLa cells. VINCULIN serves as a loading control. **E** A scheme of a fragment of *MBNL1* pre-mRNA with a  depicted position of binding of an antisense oligonucleotide (SSO-e7) complementary to a region across the 3’ss of e7 marked in red. **F** Representative gels and calculations of RT-PCR analyses of *MBNL1* e5 and e7 upon SSO-e7 treatment in HSkM cells. Data represent the mean PSI values (*n* = 3). Statistical significance was calculated in reference to SSO-Ctrl using Student’s *t* test; *ns* nonsignificant, ∗∗∗ for *P* < 0.001. **G** Representative western blot analyses show changes in distribution of MBNL1 protein isoforms upon SSO-e7 treatment in HSkM. Numbers 40–43 correspond to a molecular mass of the MBNL1 proteins and refer to* MBNL1* isoform pre-mRNAs illustrated in **C**. VINCULIN serves as a loading control. **H** Total level of MBNL1 upon SSO-e7 treatment in HSkM, as defined by western blotting and normalized to VINCULIN. Data represent the mean values ± SD (*n* = 3). Statistical significance was calculated in reference to SSO-Ctrl using Student’s *t* test; *ns* nonsignificant. **I** Representative gels and calculations of RT-PCR analyses of AS events shared by MBNL1 and DDX5,17 upon SSO-e7 treatment in HSkM. Six out of 17 analyzed events (*PICALM* e18,* SPTAN1* e23*, KTN1* e40, *CLASP1* e25, *CSDA* e6, *ACAD*10 e6) were sensitive to SSO-e7 treatment and showed splicing changes that mimicked those observed in DDX5,17-depleted myoblasts (Fig. 2H, Suppl. Figure S2B). The left and right columns correspond to AS events sensitive and nonresponsive to SSO-e7 treatment, respectively. Data represent the mean PSI values (*n* = 3). Statistical significance was calculated in reference to SSO-Ctrl using Student’s *t* test; *ns* nonsignificant, ∗∗ for *P* < 0.01, ∗∗∗ for *P* < 0.001

RNA-seq datasets retrieved from two experiments and two exon array analyses conducted in DDX5 KD or DDX5,17 KD cell lines [37, 39] showed a considerable switch in the ratio of *MBNL* splicing isoforms carrying alternative exons 5 and 7 (Fig. 3A, Suppl. Table S2). The association between DDX5,17 expression and splicing of *MBNL1* exons 5 and 7 was also noticed in different human and mouse tissues (Suppl. Figure S3B-E). To support these observations, we checked the status of all splicing isoforms in our HeLa and HSkM cell models with DDX5,17 depletion. RT-PCR analyses revealed significant exclusion of *MBNL1* e5 and e7 and *MBNL2* e5 upon DDX5,17 KD, in all tested cellular models (Fig. 3B, Suppl. Figure S4A-D). The *MBNL1* alternative e3 and e8 remained unchanged while *MBNL1* e1 was significantly excluded elevating a level of a minor *MBNL1* isoform (Suppl. Figure S4A-D). Consistently to changes in alternative splicing of *MBNL1* transcript, we detected an increase in shorter MBNL1 protein isoforms in DDX5,17 KD HeLa and HSkM cells (Fig. 3C, D, Suppl. Figure S4E, F). As expected, the DDX5,17-dependent exclusion of *MBNL1* e5, which encodes a bipartite nuclear localization signal, led to slight changes in the subcellular localization of MBNL1, as a twofold increase of MBNL1 was detected in the cytoplasm upon DDX5,17 depletion (Suppl. Figure S4G).

Previous studies showed the RNA substrate-dependent splicing activity of MBNL1 isoforms differing in e7 encoded amino-acid tract in normal and cancer cells [8, 9, 25, 31, 72]. Based on this finding, we hypothesized that *MBNL1* e7 exclusion enhances the MBNL1-regulatory activity towards shared AS events upon DDX5,17 loss. Thus, we induced a targeted *MBNL1* + e7 to − e7 isoform switch in HSkM cells using a previously described 2'-*O*-methoxyethyl phosphonothioate (2′MOE-PS) splice-switching antisense oligonucleotide complementary to the 3’ss of *MBNL1* e7 (SSO-e7) [7] (Fig. 3E). RT-PCR and western blot analyses showed robust and specific exclusion of e7 at the RNA and protein levels with no effect of SSO-e7 on the splicing of other *MBNL1* exons (Fig. 3F, G, Suppl. Figure S4H). Importantly, the total protein level of MBNL1 also remained unchanged (Fig. 3H). Next, we studied the splicing patterns of a set of AS events shared by MBNL1 and DDX5,17. Having analyzed 17 AS events with two distinguished splicing isoforms, we observed significant changes in the splicing patterns of 7 events (Fig. 3I, Suppl. Figure S2E). The splicing direction of 6 out of these 7 events mimicked that observed in DDX5,17-depleted myoblasts; however, the strength of splicing changes are lower.

Collectively, this evidence demonstrates that the absence of DDX5,17 does not impact MBNL1,2 levels. DDX5,17 affect MBNL1 splicing activity by changing the ratio of MBNL1 splicing isoforms. However, only a subset of shared AS events was subjected to the identified pathway (35%), and the splicing changes affected by the *MBNL1* + e7 to − e7 switch were statistically significant but relatively low.

### DDX5,17 may affect MBNL-dependent AS through interfering in MBNL-RNA complexes

**Fig. 4:**
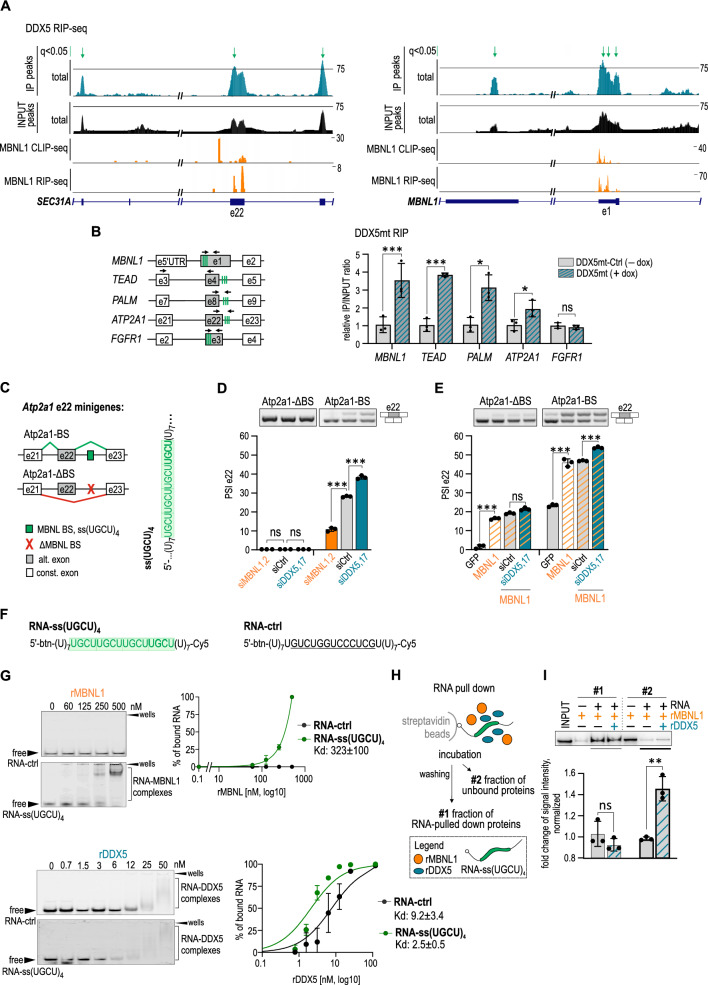
DDX5,17 affect MBNL-dependent AS perhaps through interfering in MBNL-RNA complexes. **A** DDX5 RIP-seq showing a selected region of *SEC31A* and *MBNL1*. IP track (turquoise) and INPUT track (black) show reads coverage of DDX5 RIP-seq and input sequencing, respectively. Significant DDX5 enriched regions are marked with green arrows [*q* < 0.05 calculated by MACS as false discovery rate (FDR)]. DDX5 RIP-seq tracks are presented with fixed vertical viewing range. The coverage by 75 reads is indicated by the black horizontal line. MBNL1 binding sites are indicated by CLIP-seq and RIP-seq peaks and marked with orange tracks. **B** A scheme presenting fragments of analyzed transcripts in DDX5mt RIP, with indicated MBNL-dependent AS events marked with grey boxes, potential MBNL-binding sites with green boxes and location of primers used in qPCR marked with black arrows (*left*). RT-qPCR analyses of selected transcripts in DDX5mt RIP (*right*). Data represent the mean values ± SD (*n* = 3). Statistical significance was calculated in reference to MCF7 DDX5mt cells without doxycycline induction used as a control, by applying Student’s *t* test; *ns* nonsignificant, ∗∗ for *P* < 0.01, ∗∗∗ for *P* < 0.001. **C** A scheme of two *Atp2a1* e22 splicing minigenes, Atp2a1-BS and Atp2a1-ΔBS, with or without MBNL-binding sites, respectively (*left*). The RNA sequence containing MBNL-binding sites embedded within an unstructured region composed of 7 uridines (ss; single stranded; ss(UGCU)_4_) and located within intron 22 within Atp2a1-BS minigene [31]; MBNL BS, MBNL-binding sites; MBNL ΔBS, deletion of MBNL-binding sites; alt exon, alternative exon; const. exon, constitutive exon. **D** Representative gels and calculations of RT-PCR analyses show a strength of e22 inclusion in *Atp2a1* e22 minigenes in DDX5,17 or MBNL1,2 depleted cells compared to that in control HeLa cells. Data represent the mean PSI values ± SD (*n* = 3). Statistical significance was calculated in reference to siCtrl; *ns* nonsignificant, ∗∗∗ for *P* < 0.001. **E** Representative gels and calculations of RT-PCR analyses of the *Atp2a1* e22 minigenes’ splicing response to transiently expressed GFP-MBNL1 in DDX5,17-depleted cells compared to control HeLa cells. Data represent the mean PSI values ± SD (*n* = 3). Statistical significance was calculated in reference to siCtrl using Student’s *t* test; *ns* nonsignificant, ∗∗∗ for *P* < 0.001. **F** 5′-biotin labelled and 3′-Cyanine 5 labelled short RNA sequences with either four MBNL-binding sites, ss(UGCU)_4_, marked in green boxes or randomly distributed U, G, C nucleotides, highlighted, which we used in the following EMSA and RNA pull down assays (**G**–**I**). **G** Results of EMSA showing the interaction between RNAs and recombinant proteins, rMBNL1 or rDDX5, at indicated concentrations. Representative native polyacrylamide gels showing complex formation between studied RNAs and proteins (*left*). Quantification of EMSA results based on the decline of free RNA signal in favor of forming RNA–protein complexes (*right*). *n* = 3. For each experiment dissociation constant, *K*_d_ [nM], was calculated. **H** A short pipeline of RNA pull down assay indicating two collected fractions. Fraction #1contained RNA pulled down proteins whereas fraction #2 contained unbound proteins. **I** Western blot analyses and quantification of RNA pull down assay showing an elevated level of unbound rMBNL1 in the presence of  rDDX5 in the reaction. Other analyzed samples on the gel constitute INPUT and rMBNL1 incubated with beads only (no RNA). Data represent the mean values ± SD (*n* = 3). Statistical significance was calculated in reference to a control reaction with only RNA and rMBNL using Student’s *t* test; *ns* nonsignificant, ∗∗ for *P* < 0.01

To test whether the RNA helicases directly bind to MBNL1 target transcripts containing shared events we retrieved publicly available data from DDX5 RNA-immunoprecipitation followed by next-generation sequencing (RIP-seq) conducted in RH30 cells [73]. We found significant enrichment of majority of those RNAs in an immunoprecipitated fraction compared to an input (Suppl. Figure S5A, Suppl. Table S2). For instance, we found significant peaks located in a region of *SEC31A* and *MBNL1* transcripts which are also bound by MBNL1 according to our previously published RIP-seq and CLIP-seq data (MIB.amu.edu.pl) (Fig. 4A) [9].

We further performed native RIP in an MCF7 cellular model with doxycycline induced expression of HA-tagged DDX5 mutant (DDX5mt) which has RNA binding activity but lacks helicase activity, in order to check whether DDX5 can directly bind a region carrying MBNL-binding sites. Having performed RT-qPCR analyses for a few MBNL1 targets by amplifying regions containing potential MBNL-binding sites we found enrichment of some mRNAs and pre-mRNAs including *MBNL1*, *PALM, TEAD1*, *ATP2A1* (Fig. 4B, Suppl. Figure S5B-D). These observations suggest that binding of DDX5mt to studied RNAs occurs in an ATP-independent manner.

These findings prompted us to ask whether DDX5,17 may have a direct effect on the functional interaction between MBNL1,2 and RNA *cis*-regulatory elements or work independently by cooperating with other RBPs. The latter motion was investigated in the next chapter.

First, we applied an indirect approach utilizing AS minigenes representing shared AS events with either MBNLs-induced exon inclusion (e_ON) or exclusion (e_OFF). *Atp2a1* e22 splicing minigenes (e_ON) carry either a single-stranded tract of four UGCU-tetranucleotides (ss(UGCU)_4_; Atp2a1-BS) which is a strong RNA-regulatory element bound by MBNL1,2, or a deletion of this region (Atp2a1-ΔBS) (Fig. 4C) [31]. HeLa cells were transfected with these minigenes with concomitant depletion of DDX5,17 or MBNL1,2 and the *Atp2a1* e22 splicing pattern was measured by RT-PCR (Suppl. Figure S5E). Our analyses showed that Atp2a1-ΔBS minigene was not sensitive to silencing of the helicases, contrary to Atp2a1-BS (Fig. 4D). In the latter, DDX5,17 depletion increased e22 inclusion by nearly 15% in comparison to the control level and in a direction opposite to MBNL1,2 silencing. Due to the fact that MBNL1 and MBNL2 levels in Hela cells are not very high we asked whether a depletion of helicases will further enhance MBNL1 effect on e22 splicing in cells with MBNL1 overexpression and whether this effect will be MBNL-binding site dependent. RT-PCR analyses showed that depletion of DDX5,17 had no effect on splicing of Atp2a1-ΔBS minigene in cells with overexpressed MBNL1 (Fig. 4E, Suppl. Figure S5F). Contrarily, inclusion of e22 in Atp2a1-BS minigene was increased upon MBNL1 overexpression and this effect was further enhanced upon DDX5,17 depletion. We tested another minigene carrying *MBNL1* e5 (ex_OFF) for its sensitivity to siDDX5,17 and simultaneous expression of MBNL1 and obtained consistent results (Suppl. Figure S5G-L).

To elucidate whether siDDX5,17-imposed splicing changes of studied minigenes result from the helicases and MBNLs competing for the same RNA region, we tested binding affinity of one of them, recombinant DDX5 (rDDX5) to two RNA sequences. The first RNA contained MBNL-binding sites, RNA-ss(UGCU)_4_, while a negative control, RNA-ctrl, carried randomly distributed U, G, C nucleotides in place of the (UGCU)_4_ motif (Fig. 4F). Both RNAs were conjugated at the 3′ end with Cyanine 5 which allows for fluorescence detection of RNA–protein complexes in a polyacrylamide gel and 5’end biotinylated for a subsequent RNA pull down assay. Using an in vitro electrophoretic mobility shift assay (EMSA) we showed that rDDX5 binds to both RNAs with a slightly higher preference to the MBNL-recognized RNA (Fig. 4G). Interestingly, lack of a defined band corresponding to RNA-rDDX5 complexes could indicate that formed complexes have a rather transient character and the protein quickly dissociates from RNA. To study a competition between the helicases and MBNL1 for binding to the same RNAs we performed RNA pull down assay. RNA-ss(UGCU)_4_ was first incubated with either rMBNL1 or mixture of rMBNL1 and rDDX5 and then the proteins were pulled down on streptavidin beads constituting fraction #1 (Fig. 4H). Fraction #2 corresponded to a sample collected after incubation with the beads, which contained unbound proteins. Immunoblotting of the pulled down fraction #1 did not show a statistically significant difference in rMBNL1 level between these two conditions but fraction #2 contained significantly more unbound rMBNL1 in the presence of rDDX5 in the reaction (Fig. 4I). It indicates that rDDX5 competes with rMBNL1 for binding to RNA-ss(UGCU)_4_ but under these conditions it is a weak competitor.

The obtained results indicate that DDXs interfere with MBNLs activity by most likely hindering the functional interaction between MBNL1 and RNA targets. DDXs may affect MBNL-RNA complex formation and/or trigger MBNL dissociation through changes in RNA structural arrangement or protein–protein interactions.

### RNA helicases modulate the AS of certain shared events independent of MBNL1,2 activity

**Fig. 5:**
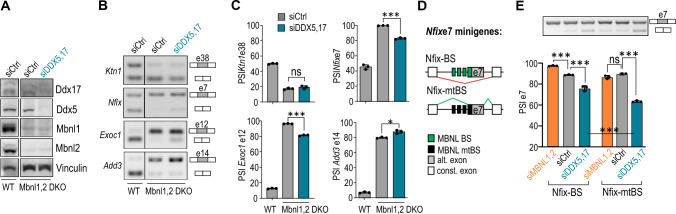
DDX5,17 modulate AS of certain shared events independently of MBNL1,2. **A** Representative western blot analyses show protein levels in wild-type (WT) and Mbnl1,2 DKO MEFs upon Ddx5,17 depletion. Vinculin serves as a loading control. Blot images of all samples and calculated protein levels are shown in Suppl. Figure S6A. All samples derive from the same experiment and the blots were processed in parallel. **B** Representative gels of RT-PCR analyses of MBNL-regulated events whose splicing direction was either unchanged in DDX5,17 KD and Mbnl1,2 DKO cells (*Ktn1* e38) or significantly deviated compared to that in control cells (*Nfix* e7, *Exoc1* e12, *Add3* e14). The image presents juxtaposed lanes that were non-adjacent on the gel. The lanes separation is delineated by a black separation. All samples derive from the same experiment and the bands were processed in parallel. **C** Calculation of RT-PCR analyses presented in **B**. Data represent the mean PSI values ± SD (*n* = 3). Statistical significance was calculated in reference to siCtrl using Student’s *t* test; *ns* nonsignificant, ∗ for *P* < 0.05, ∗∗∗ for *P* < 0.001. **D** A scheme of two *Nfix* e7 splicing minigenes, Nfix-BS and Nfix-mtBS, which carry MBNL-binding sites within an exon and upstream intron or their mutations, respectively [60]. MBNL BS, MBNL-binding site; MBNL mtBS, MBNL-binding sites carrying mutations; alt exon, alternative exon; const. exon, constitutive exon. **E** Representative gels and calculations of RT-PCR analyses of the *Nfix* e7 minigene splicing response in DDX5,17-depleted HeLa cells. Data represent the mean PSI values ± SD (*n* = 3). Statistical significance was calculated in reference to siCtrl using Student’s *t* test; *ns* nonsignificant, ∗∗∗ for *P* < 0.001

Thus far, we had shown that the splicing regulation of shared events mediated by DDX5,17 involves changes in the ratio of MBNL1 splicing isoforms and most likely a functional MBNL-RNA interaction. Since previous studies demonstrated RNA helicases to cooperate with several RBPs [36, 37, 49, 54], we hypothesized that other splicing factors contribute to the DDX5,17-mediated splicing regulation of shared AS events (hypothesis III). For this purpose, we performed Ddx5,17 KD in Mbnl1,2 double knock-out (DKO) mouse embryonic fibroblasts (MEFs) (Fig. 5A, Suppl. Figure S6A). Despite the loss of Mbnl1,2, we identified a group of Mbnl-dependent AS events for which Ddx5,17 KD causes a significant splicing change, including *Nfix* e7, *Exoc1* e12, and *Add3* e14 (Fig. 5B, C, Suppl. Figure S6B). This suggests that Ddx5,17 partially regulate shared events independent of Mbnl1,2. Interestingly, the absence of a change in *Ktn1* e38 splicing indicates that Ddx5,17 regulate this exon by the Mbnl1-isoform switch pathway and potentially by inhibiting the functional Mbnl-RNA interaction (Fig. 3E, I).

To support these observations, we investigated the MBNL-independent pathway of AS regulation by DDX5,17 by examining previously described wild-type and mutant *Nfix* e7 splicing minigenes (Nfix-BS, Nfix-mtBS) [60]. The wild-type Nfix-BS minigene contains several widely scattered MBNL1-binding sites in intron 6 and exon 7 (Fig. 5D). Having analyzed the splicing responses of both minigenes in HeLa cells with DDX5,17 or MBNL1,2 depletion, we noticed that e7 in Nfix-BS was sensitive to the KD of both classes of splicing regulators (Fig. 5E). As expected, mutations in MBNL-binding sites abolished the response of Nfix-mtBS e7 to MBNL1,2 KD but not DDX5,17 KD. Moreover, we noticed that Nfix-mtBS e7 was excluded to a greater extent than Nfix-BS e7, suggesting that possible competition between MBNL1 and other splicing factor(s) contributes to the DDX5,17-mediated splicing regulation.

These results support the MBNL-independent function of DDX5,17 in AS regulation of a subset of shared events. This phenomenon is presumably linked to other splicing factors that regulate shared AS events in MBNLs-independent ways. These helicases can cooperate with either MBNL antagonists or RBPs working alongside MBNLs.

## Discussion

In this study, we show a deeper insight into a modulatory role of DDX5,17 helicases in regulation of MBNL-dependent AS. Our studies indicate that a high percentage of RT-PCR-analyzed MBNL-dependent AS events (both e_ON and e_OFF) are codependent on DDX5 and DDX17 levels. The splicing of majority of these shared events changes in an opposite direction upon silencing of these two classes of RBPs. Even though the strength of splicing changes of individual events is small or moderate, it concerns a wide group of splicing events which cumulatively may have a biological effect. To date, a few other networks of interdependencies in coordinate regulation of AS between DDX5,17 and splicing factors have been described, including hnRNP F/H [37] and hnRNP A1, which were shown for a large subset of AS events, as well as RBM4 [36], hnRNP H [54] and MBNL1 itself [34], which were described just for individual events. Some were identified in the context of physiological or pathological processes [37, 38].

**Fig. 6:**
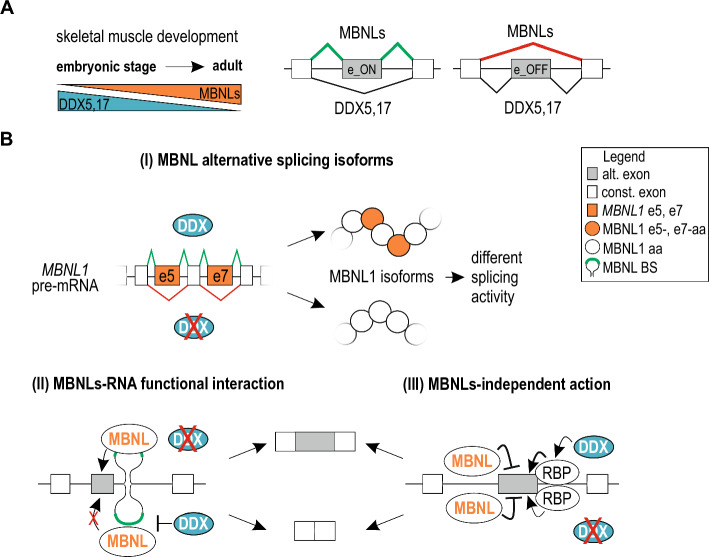
Modulatory role of DDX5,17 and its underlying pathways in MBNL-dependent AS regulation. **A** A model showing the functional modulatory impact of DDX5,17 on AS regulated by MBNL during skeletal muscle development. Opposite expression pattern of MBNLs and DDX5,17 (*left*) and an opposite effect on AS of these two classes of RBPs (*right*) in regulation of MBNL-dependent e_ON and e_OFF events may be essential determinants of the strength of splicing changes of MBNL-dependent events during development.; **B** A model representing three pathways underlying the modulatory role of DDX5,17 in MBNL-dependent AS regulation. More explanation is provided in the main text. *MBNL1* e5, e7, *MBNL1* alternative exons 5 and 7; MBNL1 e5-, e7-aa, MBNL1 e5- and e7-encoded aminoacid sequences; MBNL1 aa, aminoacid sequences encoded by other exons than e5 and e7; MBNL BS, MBNL-binding site within target RNAs

The mechanism underlying modulatory role of the helicases seems to be multilayered. Here, we identified three pathways (I-III) either dependent or independent of direct interplay between these two groups of RBPs (Fig. 6B). First, we showed that DDX5,17 regulate AS of shared events by affecting the splicing of *MBNL1* alternative exons and, consequently, the MBNL1 splicing activity (pathway I) (Fig. 3). Recent findings of our group and others have shown that MBNL1 isoforms differ in terms of their splicing properties in an RNA substrate-dependent manner [7–9, 31]. Utilized by us SSO-approach to induce the selective exclusion of e7 from the *MBNL1* transcript in human myoblast cells showed splicing changes of 1/3 of the analyzed shared AS events mimicking the direction of splicing found in DDX5,17-depleted cells. Therefore, certain splicing alterations observed by us upon depletion of helicases are perhaps a downstream effect of changes in MBNL1 isoform distribution.

Pathway II includes potential interference of the helicases in functional interactions between MBNL1,2 and its RNA targets (Fig. 6B). We showed that DDX5 binds to MBNL-dependent transcripts (RIP) and favors binding to RNA containing MBNL-binding sites (EMSA). On the functional level, the regulation of *Atp2a1* e22 splicing minigeneby endogenous or exogenous MBNL1 was enhanced on the background of DDX5,17 depletion only when MBNL-binding sites were present in a construct (Atp2a1-BS)  (Fig. 4). It indicates that DDX5,17 may directly hinder MBNL-RNA complex formation or lead to its dissociation through changes in RNA structural arrangement or by directly interacting with MBNL1 protein [51]. Laurent and others have previously described that DDX5,17 mediate functional changes in the structural arrangement of MBNL-binding sites within *TNNT2* e5, resulting in enhancement of the MBNL1 splicing effect [34]. Consistent positive DDX5,17 activity was described for coordinated AS regulation along with hnRNP A1 [38] hnRNP F/H [37] and RBM4 [36] whereas DDX5,17-dependent RNA structural changes prevented hnRNP H from binding its pre-mRNA *cis*-acting regulatory elements [54].

Pathway III describes the regulatory role of DDX5,17 independent of MBNL activity (Fig. 6B). It most likely relies on cooperation of the helicases with other RBPs. In our analyses, we identified several such shared events that were sensitive to Ddx5 and Ddx17 depletion on the background of Mbnl1,2 DKO (Fig. 5). We subsequently confirmed the mode of action using the *Nfix* e7 minigene with mutations of MBNL1-binding sites. The fact that DDX5,17 could cooperate with other RBPs that antagonize or function alongside MBNLs is consistent with previous findings showing an interplay between the helicases and other RBPs, e.g., hnRNP F/H, in the regulation of AS events elsewhere listed as MBNL targets including *Tau* e10, *ARFGAP2* e8, *PLOD2* e14, *INSR* e11, *PHKA1* e19 or *Add3* e14 [9, 36, 37, 74].

The identified sensitivity of analyzed MBNL-dependent splicing events to DDX5,17 level may rely on one of the described RNA target-dependent pathways. It is more likely, however, that for majority of events these pathways occur concomitantly and together contribute to the overall splicing decision.

Interestingly, Mbnl1 and Ddx5 are characterized by opposite expression patterns during skeletal muscle development which is supported by previous studies [21, 37] (Fig. 6A). Moreover, opposite impact of helicases on MBNL-dependent splicing affects a developmental splicing pattern of tested AS events at early stages of myogenesis (Fig. 2). It may also lead to enhanced strength of splicing changes dependent on development and affect the production of splicing isoforms distinct in space, time and biological importance. For instance, due to functional depletion of MBNLs and increased expression of helicases in myotonic dystrophy type 1 (DM1) [35, 75], the identified relation between these proteins could, to some extent, contribute to the pathomechanism of DM1. DM1 is a neuromuscular disease caused by an expanded trinucleotide tract of CTG repeats within the 3′ untranslated region of the *DMPK* gene [75]. *DMPK* mRNA is retained in the nucleus and forms nuclear inclusions along with other RBPs, mostly MBNLs, which bind an expanded CUG tract, (CUG)^exp^ [76]. DDX5,17, however, modulate the amount of these inclusions and form aggregates that colocalize with toxic CUG repeats [34]. Nothing is known whether the aggregation leads to a decrease in helicases functional level but we know that DM1-patient derived myoblasts are characterized by their elevated production [35]. In DM1-affected muscle tissues [77], we identified an increase in *DDX5,17* expression which correlated to some extent with the severity of the disease (Suppl. Figure S7A). In most severely affected samples 8% of misspliced AS events overlapped with those changed in HEK293 DDX5,17 KD cells (Suppl. Figure S7B, Suppl. Table S2). Similar results were obtained for a DM1-mouse model, HSA^LR^. Interestingly, along with decreasing ankle dorsiflexion strength in DM1 patients the expression of the helicases increased (Suppl. Figure S7C). Nevertheless nothing is known about the actual protein level of helicases in DM1 adult muscles. The resulting sequestration of MBNLs by (CUG)^exp^ leads to severe mis-splicing of their targets, which return to fetal-like isoforms [75]. In line with our results, imbalance of DDX5,17 in DM1-derived myoblasts either during muscle formation or regeneration may further misregulate MBNL-dependent splicing and disturb proper maturation of transcripts either through direct or indirect pathways. Further research focusing on this aspect would be of great value.

Since MBNL-dependent splicing sensitive to DDX5,17 level was identified by us in different cellular models, suggesting its ubiquity, we assume that this coregulatory network may be important in shaping physiological processes. For example, the level of DDX5,17 strongly differs between adult murine skeletal muscles, heart, lung and brain tissues (Suppl. Figure S3B-E). Moreover the presence of *MBNL1* isoforms with or without e7 correlates with the level of DDX5 and DDX17 production. The question arises to what extent the modulatory effect of DDX5,17 may affect MBNL-dependent transcript maturation through direct or indirect pathways and shape some processes in those tissues. Is a high level of the helicases able to attenuate the effect of MBNLs on their targets in some tissues and contribute to distinct transcriptome and proteome compared to tissues with low level of helicases? Moreover, some pathological states are characterized by altered balance between these RBPs. For instance, increased production of DDX5 and DDX17 has been linked to tumorigenesis in certain types of cancer [78, 79], while MBNL1 isoforms have been found to differently affect cancer cell viability and migration [7]. It would be of great value to investigate whether the relationship between studied RBPs characterizes and shapes tumorigenesis.

### Supplementary Information

Below is the link to the electronic supplementary material.

Supplementary file1 (PDF 2579 KB)

Supplementary file2 (XLSX 26 KB)

Supplementary file3 (XLSX 2043 KB)

## Data Availability

Data supporting this study are included within the article and supporting materials.
