# Supplementary material for: Modulatory role of RNA helicases in MBNL-dependent alternative splicing regulation
Source: Cell Mol Life Sci. 2023 Oct 26;80(11):335. doi: 10.1007/s00018-023-04927-0 (PMC10602967; doi:10.1007/s00018-023-04927-0)
Supplement: Supplementary file 1 — Supplementary file1 (PDF 2579 KB) [file 18_2023_4927_MOESM1_ESM.pdf]

# Modulatory role of RNA helicases in MBNL-dependent alternative splicing regulation

Katarzyna Taylor[1,*], Agnieszka Piasecka[1], Arkadiusz Kajdasz[2], Aleksandra Brzęk[1], Micaela Polay Espinoza[3], Cyril F. Bourgeois[3], Artur Jankowski[1], Małgorzata Borowiak[1], Katarzyna D. Raczyńska[1], Łukasz J. Sznajder[4,5], Krzysztof Sobczak[1,*]

[1]Department of Gene Expression, Institute of Molecular Biology and Biotechnology, Adam Mickiewicz University, Uniwersytetu Poznanskiego 6, 61-614 Poznań, Poland
[2]Institute of Bioorganic Chemistry, Polish Academy of Sciences, Noskowskiego 12/14, 61-704 Poznań, Poland
[3]Laboratoire de Biologie et Modelisation de la Cellule, Ecole Normale Superieure de Lyon, CNRS, UMR 5239, Inserm, U1293, Universite Claude Bernard Lyon 1, 46 allee d'Italie F-69364 Lyon, France
[4]Department of Molecular Genetics and Microbiology, Center for NeuroGenetics and the Genetics Institute, University of Florida, College of Medicine, Gainesville, FL 32610, USA
[5]Department of Chemistry and Biochemistry, University of Nevada, Las Vegas, NV 89154, USA.

[*]To whom correspondence should be addressed: Tel. 61-829-5766, ksobczak@amu.edu.pl. Correspondence may also be addressed to Tel. 61-829-5952, kksiazek@amu.edu.pl.

## SUPPLEMENTARY FIGURES

**Supplementary Figure S1.** DDX5,17 are splicing modulators of a large subset of MBNL-dependent AS events in Hela cell models.

**Supplementary Figure S2.** DDX5,17 affect the splicing of developmentally-regulated and MBNL-dependent AS events in human and mice muscle cells.

**Supplementary Figure S3.** DDX5,17 depletion does not affect MBNL1,2 protein levels, but their expression levels in different mice and human tissues associate with the ratio of *MBNL1* splicing isoforms.

**Supplementary Figure S4.** DDX5,17 depletion affect the ratio of mRNA and protein MBNL1,2 splicing isoforms in HeLa and HSkM cell models.

**Supplementary Figure S5.** DDX5,17 affect MBNL-dependent AS through most likely interfering in MBNL-RNA complexes.

**Supplementary Figure S6.** DDX5,17 modulate AS of certain shared events independent of MBNL1,2.

**Supplementary Figure S7.** Expression levels of DDX5 and DDX17 in DM1-affected muscle tissues associate with the disease severity.

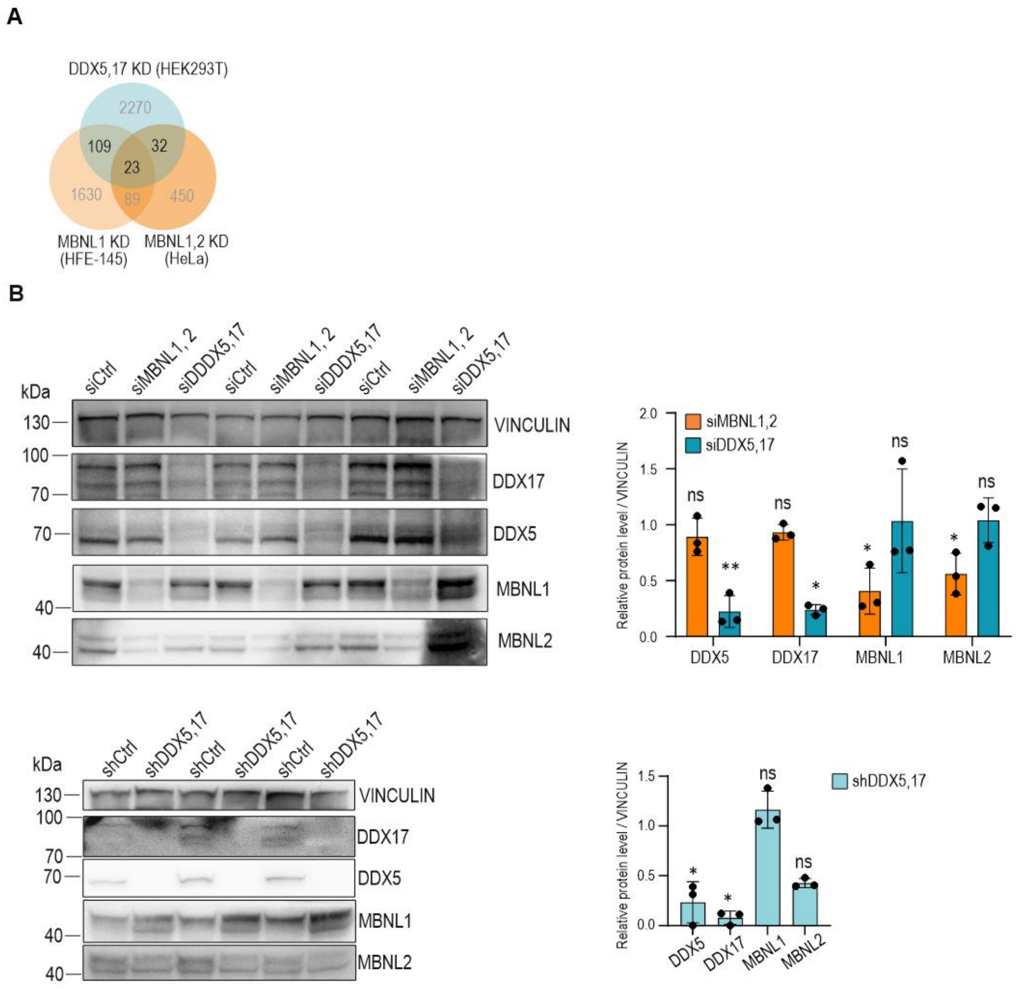


**Supplementary Figure S1**

C

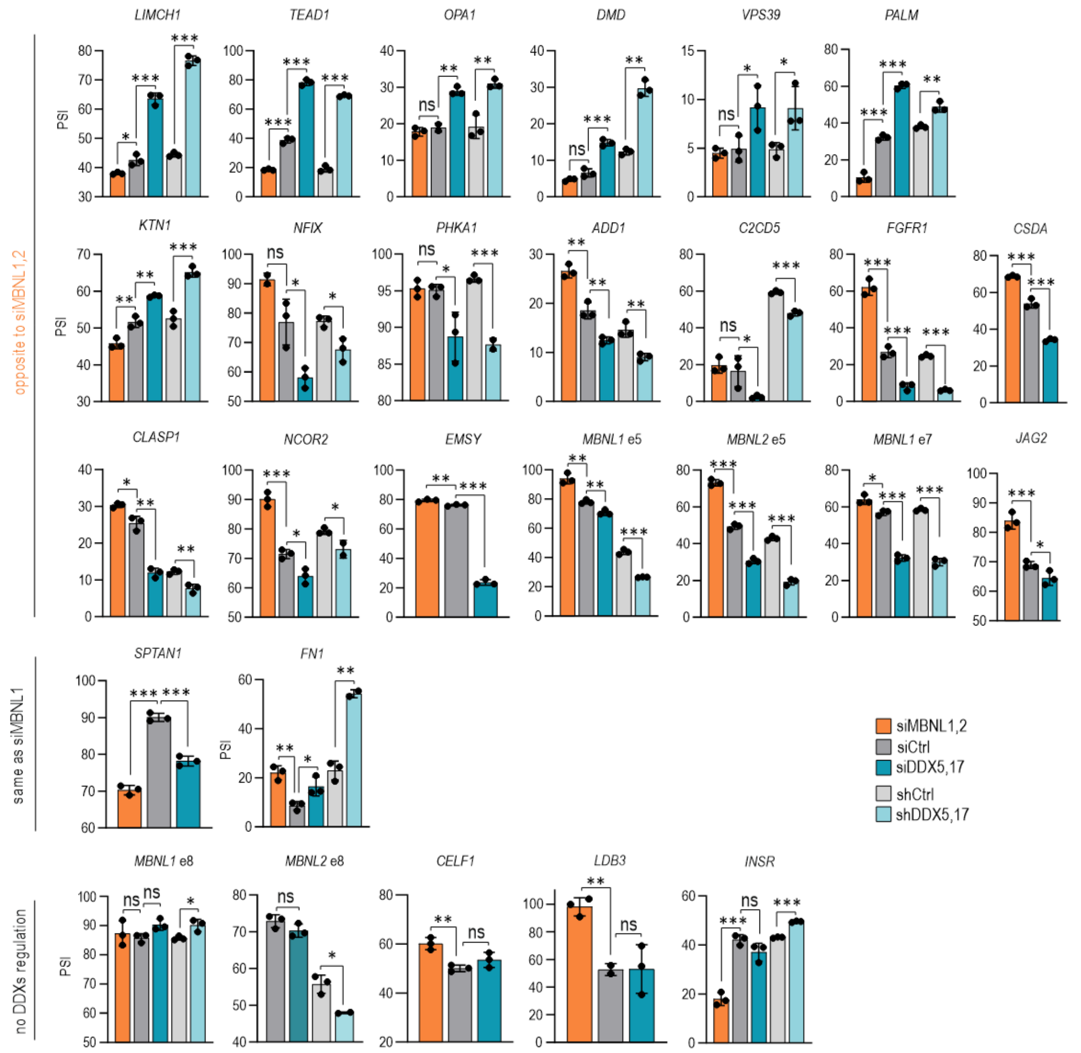
opposite to siMBNL1,2
same as siMBNL1
no DDXs regulation
PSI
LIMCH1
TEAD1
OPA1
DMD
VPS39
PALM
KTN1
NFIX
PHKA1
ADD1
C2CD5
FGFR1
CSDA
CLASP1
NCOR2
EMSY
MBNL1 e5
MBNL2 e5
MBNL1 e7
JAG2
SPTAN1
FN1
MBNL1 e8
MBNL2 e8
CELF1
LDB3
INSR
siMBNL1,2
siCtrl
siDDX5,17
shCtrl
shDDX5,17

D

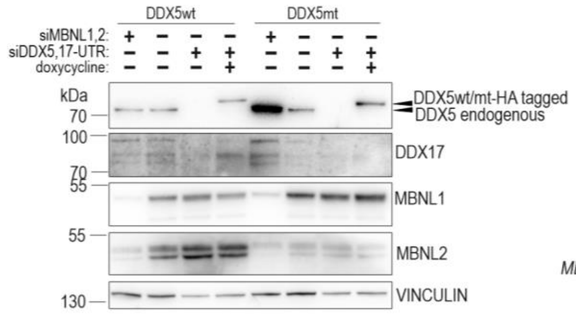
DDX5wt
DDX5mt
siMBNL1,2:
siDDX5,17-UTR:
doxycycline:
kDa
70
100
70
55
55
130
DDX5wt/mt-HA tagged
DDX5 endogenous
DDX17
MBNL1
MBNL2
VINCULIN

E

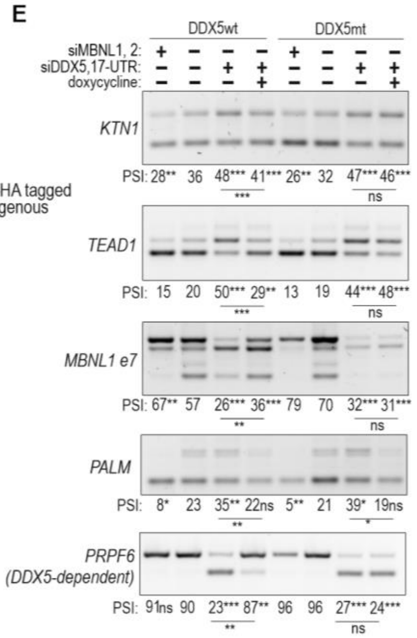
DDX5wt
DDX5mt
siMBNL1, 2:
siDDX5,17-UTR:
doxycycline:
KTN1
PSI: 28** 36 48*** 41*** 26** 32 47*** 46***
TEAD1
PSI: 15 20 50*** 29** 13 19 44*** 48***
MBNL1 e7
PSI: 67** 57 26*** 36*** 79 70 32*** 31***
PALM
PSI: 8* 23 35** 22ns 5** 21 39* 19ns
PRPF6
(DDX5-dependent)
PSI: 91ns 90 23*** 87** 96 96 27*** 24***

**Supplementary Figure S1**

**DDX5,17 are splicing modulators of a large subset of MBNL-dependent AS events in Hela cell models.**

A) The Venn diagram shows the number of AS events which changed at the global level in MBNL1,2 KD or DDX5,17 KD cells. Computed ΔPSI values from RNA-seq AS analyses were compared between DDX5,17 KD HEK293T cells (n = 3; [1]), MBNL1,2 KD HeLa cells (n = 1; [2]) and MBNL1 KD HFE-145 cells (n = 3; [3]). All AS events met the criteria of FDR ≤ 0.05 and |ΔPSI| ≥ 10. Note that differences in cell models and experimental procedures introduce a bias to this AS analyses.

B) Western blot analyses of DDX5, DDX17, MBNL1 and MBNL2 levels in HeLa cells upon knockdown of DDX5,17 or MBNL1,2 with siRNA or shRNA. Immunoblot images of western blot with VINCULIN serving as a loading control (*left*). Calculations of western blot (*right*). Data represent the mean values +/- SD (n = 3). Statistical significance was calculated in reference to control (siCtrl or shCtrl) using Student's t-test; ns, nonsignificant, ∗ for $P < 0.05$, ∗∗ for $P < 0.01$).

C) RT-PCR analyses of AS of 27 randomly selected MBNL-dependent AS events in two HeLa models upon DDX5,17 silencing with either siRNA or shRNA. Data represent the mean PSI values +/- SD (n = 3). Statistical significance was calculated in reference to control (siCtrl or shCtrl) using Student's t-test; ns, nonsignificant, ∗ for $P < 0.05$, ∗∗ for $P < 0.01$, ∗∗∗ for $P < 0.001$. For the majority of events the direction of splicing changes was opposite between cells transfected with siMBNL1,2 and those transfected with siDDX5,17.

D) Representative western blot of DDX5, DDX17, MBNL1 and MBNL2 levels in MCF7 cells upon knockdown of DDX5,17 and simultaneous doxycycline induced expression of DDX5-HA wild type or DDX5-HA mutant without a helicase activity. VINCULIN serves as a loading control.

E) RT-PCR splicing analyses of shared AS events (*KTN1*, *TEAD1*, *MBNL1* e7, *PALM*) and a DDX5-dependent event as a control (*PRPF6*) upon DDX5,17 silencing and inducible expression of HA-tagged DDX5 wildtype or DDX5 mutant in MCF7 cells. DDX5 mutant does not have a helicase activity. Data represent the mean PSI values +/- SD (n = 3). Statistical significance was calculated in reference to control (siCtrl) using Student's t-test; ns, nonsignificant, ∗ for $P < 0.05$, ∗∗ for $P < 0.01$, ∗∗∗ for $P < 0.001$.

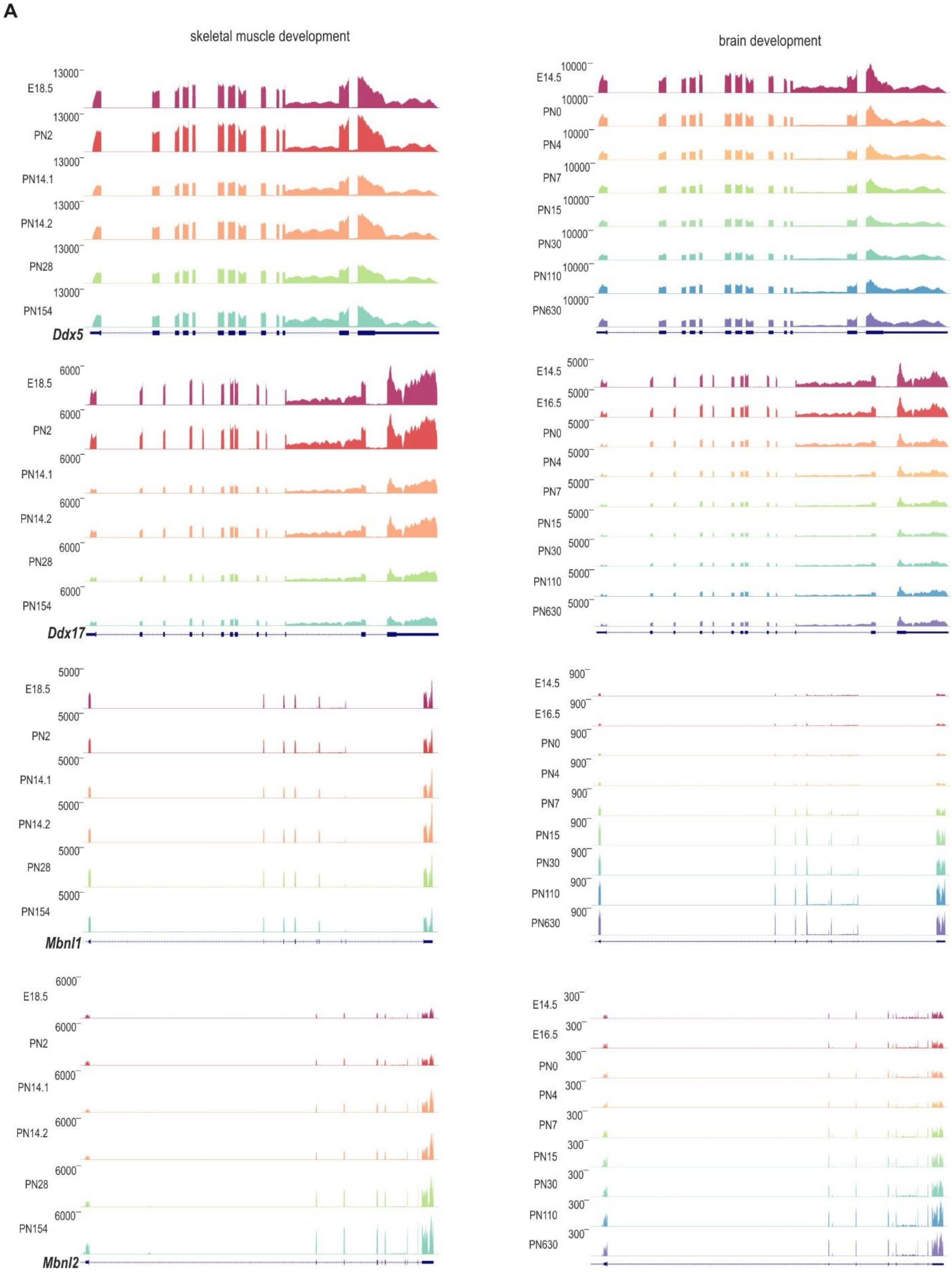


**Supplementary Figure S2**

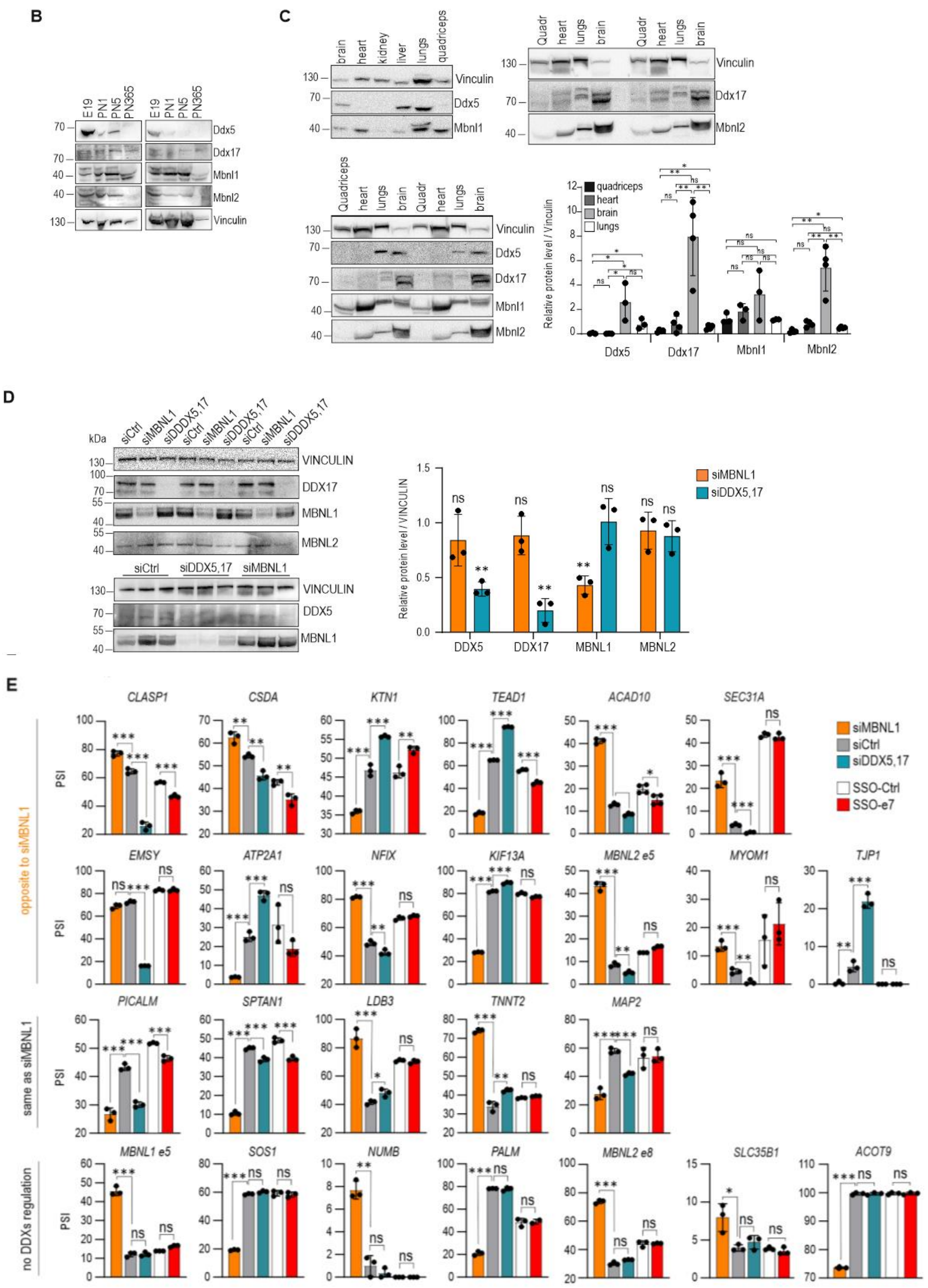


**Supplementary Figure S2**

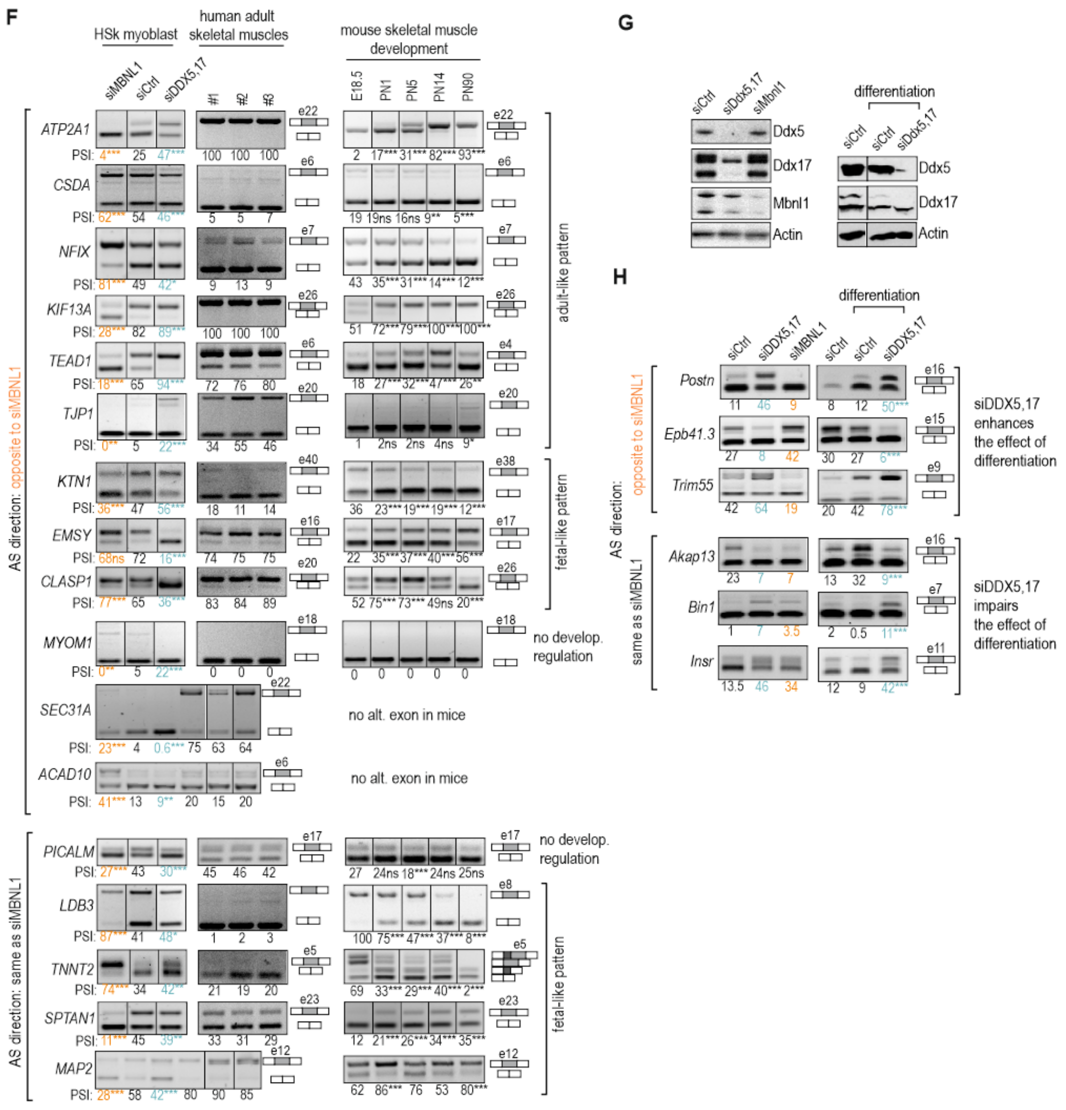


**Supplementary Figure S2**

**DDX5,17 affect the splicing of developmentally-regulated and MBNL-dependent AS events in human and mice muscle cells.**

A) Genome browser view (mm10) showing *Mbnl1*, *Mbnl2*, *Ddx5* and *Ddx17* gene expression at several stages of skeletal muscles (n = 1; [4]) and the brain (n = 2; [5]) development. The absolute read counts for each sample are indicated on the y axis.

B) Western blot analyses of DDX5, DDX17, MBNL1 and MBNL2 levels in murine tissues in 4 developmental stages (E19, PN1, PN5, adult). Vinculin serves as a loading control; n = 2.

C) Western blot analyses of DDX5, DDX17, MBNL1 and MBNL2 levels in murine tissues. Immunoblot images of western blot with Vinculin serving as a loading control (*left*). Each loading control corresponds to the proteins below it. All samples derive from the same experiment and the blots were processed in parallel. Calculations of western blot (*right*). Data represent the mean values +/- SD (n = 3 or 4). Statistical significance was calculated using Student's t-test; ns, nonsignificant, $*$ for $P < 0.05$, $**$ for $P < 0.01$.

D) Western blot analyses of DDX5, DDX17, MBNL1 and MBNL2 levels in HSkM cells upon knockdown of DDX5,17 or MBNL1,2. Immunoblot images of western blot with VINCULIN serving as a loading control (*left*). Each loading control corresponds to the proteins below it. All samples derive from the same experiment and the blots were processed in parallel. Calculations of

western blot (*right*). Data represent the mean values +/- SD (n = 3). Statistical significance was calculated in reference to a control, siCtrl, using Student's t-test; ns, nonsignificant, ∗∗ for $P < 0.01$.

E) RT-PCR analyses of MBNL-regulated AS events in HSkM upon DDX5,17 depletion or SSO-e7 treatment. Data represent the mean PSI values +/- SD (n = 3). Statistical significance was calculated in reference to control (siCtrl or SSO-Ctrl) using Student's t-test; ns, nonsignificant, ∗ for $P < 0.05$, ∗∗ for $P < 0.01$, ∗∗∗ for $P < 0.001$.

F) Representative gels and calculations of RT-PCR analyses of AS events shared by MBNL1 and DDX5,17 in siRNA treated HSkM in association with the splicing pattern in adult human skeletal muscles and splicing transition of these events in the course of 5 developmental stages of murine skeletal muscles. Note that the AS directions in MBNL1 KD and DDX5,17 KD cells were mainly opposite. The splicing pattern in DDX5,17 KD cells mostly corresponded to the adult-like splicing isoforms when compared to the isoforms in human and mice adult skeletal muscles. Adult human skeletal muscles are derived from individuals at the age of 24, 43 and 76, respectively. The image presents juxtaposed lanes that were non-adjacent on the gel. The lanes separation is delineated by a black separation. All samples derive from the same experiment. Data represent the mean PSI values +/- SD (n ≥ 3). Statistical significance was calculated in reference to control (siCtrl or E18.5) using Student's t-test; ns, nonsignificant, ∗ for $P < 0.05$, ∗∗ for $P < 0.01$, ∗∗∗ for $P < 0.001$.

G) Representative western blot analyses of protein levels of Ddx5, Ddx17 and Mbnl1 protein levels in siRNA-treated C2C12 cells. Actin served as a loading control. The image presents juxtaposed lanes that were non-adjacent in the blot. The lanes separation is delineated by a black separation. All samples derive from the same experiment.

H) Representative gels and calculations of RT-PCR analyses of developmentally regulated AS events shared by Mbnl1 and Ddx5,17 in Mbnl1 KD and Ddx5,17 KD C2C12 cells under control conditions (*left*) and in the course of C2C12 differentiation (*right*). Statistical significance was calculated in reference to control differentiation (siCtrl) using Student's t-test; ∗∗∗ for $P < 0.001$.

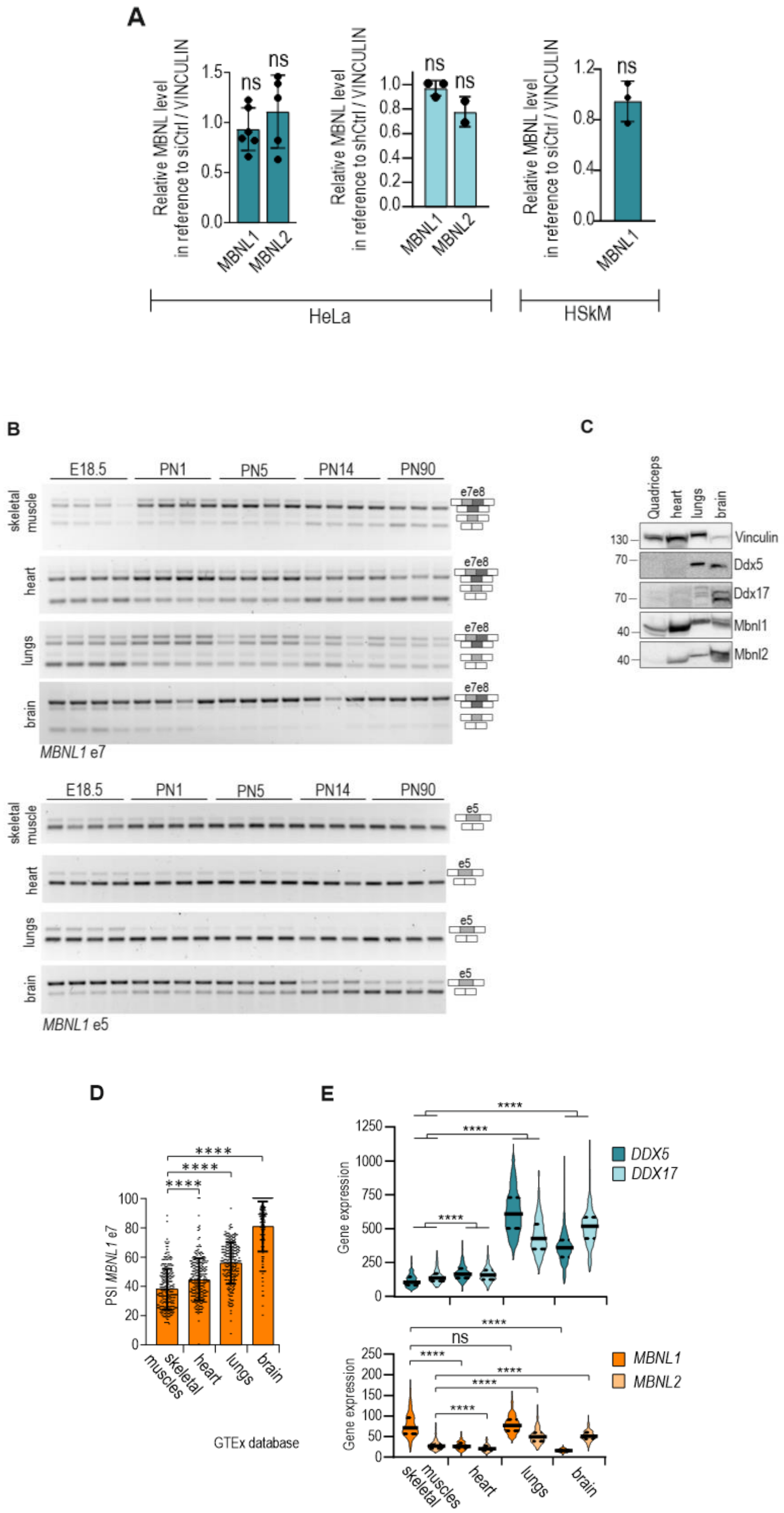


**Supplementary Figure S3**

**Supplementary Figure S3**

**DDX5,17 depletion does not affect MBNL1,2 protein levels, but their expression levels in different mice and human tissues associate with the ratio of *MBNL1* alternative splicing isoforms.**

A) Total protein levels of MBNL1 and MBNL2 upon siRNA or shRNA induced DDX5,17 depletion compared to those in control HeLa cells or HSkM, normalized to GAPDH. Data represent the mean values +/- SD. Statistical significance was calculated in reference to siCtrl using Student's t-test; ns, nonsignificant.
B) RT-PCR analyses of *Mbnl1* e5 and e7 in mouse skeletal muscles, the heart, lungs and the brain across 5 developmental stages (n = 4).
C) Representative western blot analyses of Ddx5, Ddx17, Mbnl1 and Mbnl2 protein levels in skeletal muscles, the heart, lungs and the brain of adult mice (PN365).
D) AS profile of *MBNL1* e7 in human skeletal muscles, the heart, lungs and the brain calculated at a global level based on the Genotype-Tissue Expression (GTEx) database and exon-exon junction data. Data represent the mean values +/- SD. Statistical significance was determined by the Mann-Whitney test; ∗∗∗∗ for $P < 0.0001$.
E) Gene expression profiles of *DDX5*, *DDX17*, *MBNL1* and *MBNL2* in human skeletal muscles, the heart, lungs and the brain calculated at a global level based on the Genotype-Tissue Expression (GTEx) database. Statistical significance was determined by the Mann-Whitney test; ns, nonsignificant; ∗∗∗∗ for $P < 0.0001$.

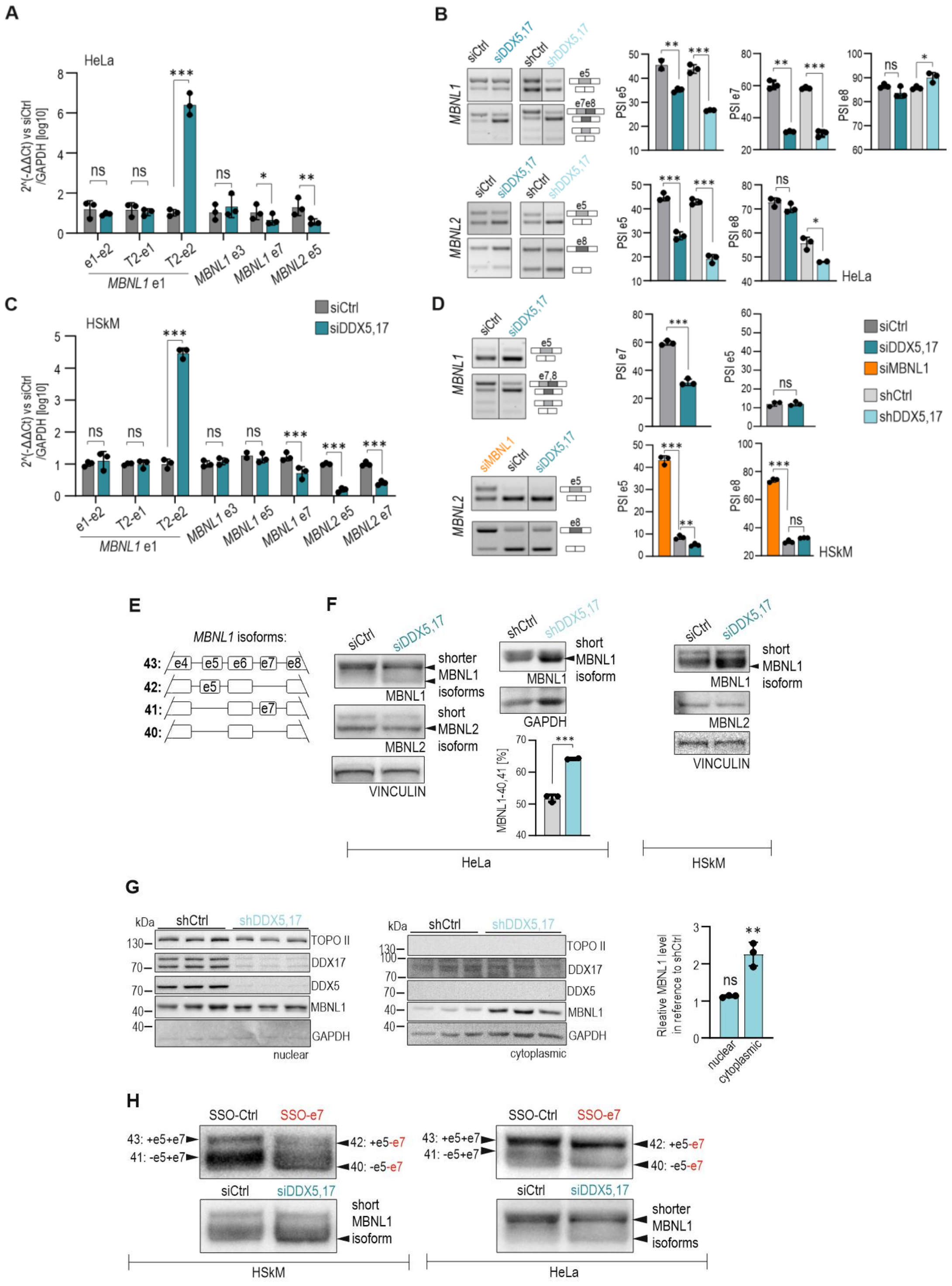


**Supplementary Figure S4**

**Supplementary Figure S4**

**DDX5,17 depletion affect the ratio of mRNA and protein MBNL1,2 splicing isoforms in HeLa and HSkM cell models.**

A) RT-qPCR splicing analyses of *MBNL1* and *MBNL2* AS events in HeLa cells upon DDX5,17 silencing. Data represent the mean values +/- SD (n = 3). Statistical significant was determined by Student's t-test; ns, nonsignificant, ∗ for $P < 0.05$, ∗∗ for $P < 0.01$, ∗∗∗ for $P < 0.001$. *MBNL1* e1 inclusion was assessed based on two pairs of primers complementary to either *MBNL1* e1 and e2 or *MBNL1* eT2, carrying transcription start site 2, and e1. *MBNL1* e1 skipping was assessed based on the primers complementary to *MBNL1* eT2 and e2. Note, that depletion of DDX5,17 promotes exclusion of *MBNL1* e1 and enrichment of a minor *MBNL1* isoform lacking e1 (primers T2-e2).

B) Representative gels and calculations of RT-PCR splicing analyses of *MBNL1* and *MBNL2* AS events in HeLa cells upon siRNA- or shRNA-induced silencing of DDX5,17. Data represent the mean PSI values +/- SD (n = 3). The image presents juxtaposed lanes that were non-adjacent on the gel. The lanes separation is delineated by a black separation. Statistical significant was determined by Student's t-test; ns, nonsignificant, ∗ for $P < 0.05$, ∗∗ for $P < 0.01$, ∗∗∗ for $P <$ 0.001.

C) As in S4A but for HSkM.

D) As in S4B but for HSkM.

E) Graphical representation of distinct *MBNL1* isoform pre-mRNAs differing in the presence of AS events.

F) Representative western blot analyses show distributions of MBNL1 and MBNL2 protein isoforms in DDX5,17 KD Hela cells or HSkM compared to those in a control. VINCULIN and GAPDH served as loading controls. Data for shRNA represent the mean +/- SD (n = 3). Statistical significant was determined by Student's t-test; ∗∗∗ for $P < 0.001$.

G) Representative western blot analyses show subcellular protein fractionation conducted after 5 days of doxycycline treatment of a stable Hela cell line expressing shRNAs against DDX5,17 (*left*). Nuclear topoisomerase II, TOPOII and cytosolic GAPDH were monitored to assess the purity of the subcellular fractions. Calculation of western blot data normalized to TOPO II or GAPDH (*right*). Data represent the mean values +/- SD (n = 3). Statistical significance was calculated in reference to shCtrl using Student's t-test; ns, nonsignificant, ∗∗ for $P < 0.01$.

H) Representative western blot analyses show distributions of MBNL1 protein isoforms in HeLa or HSkM treated with SSO-e7 or siDDX5,17.

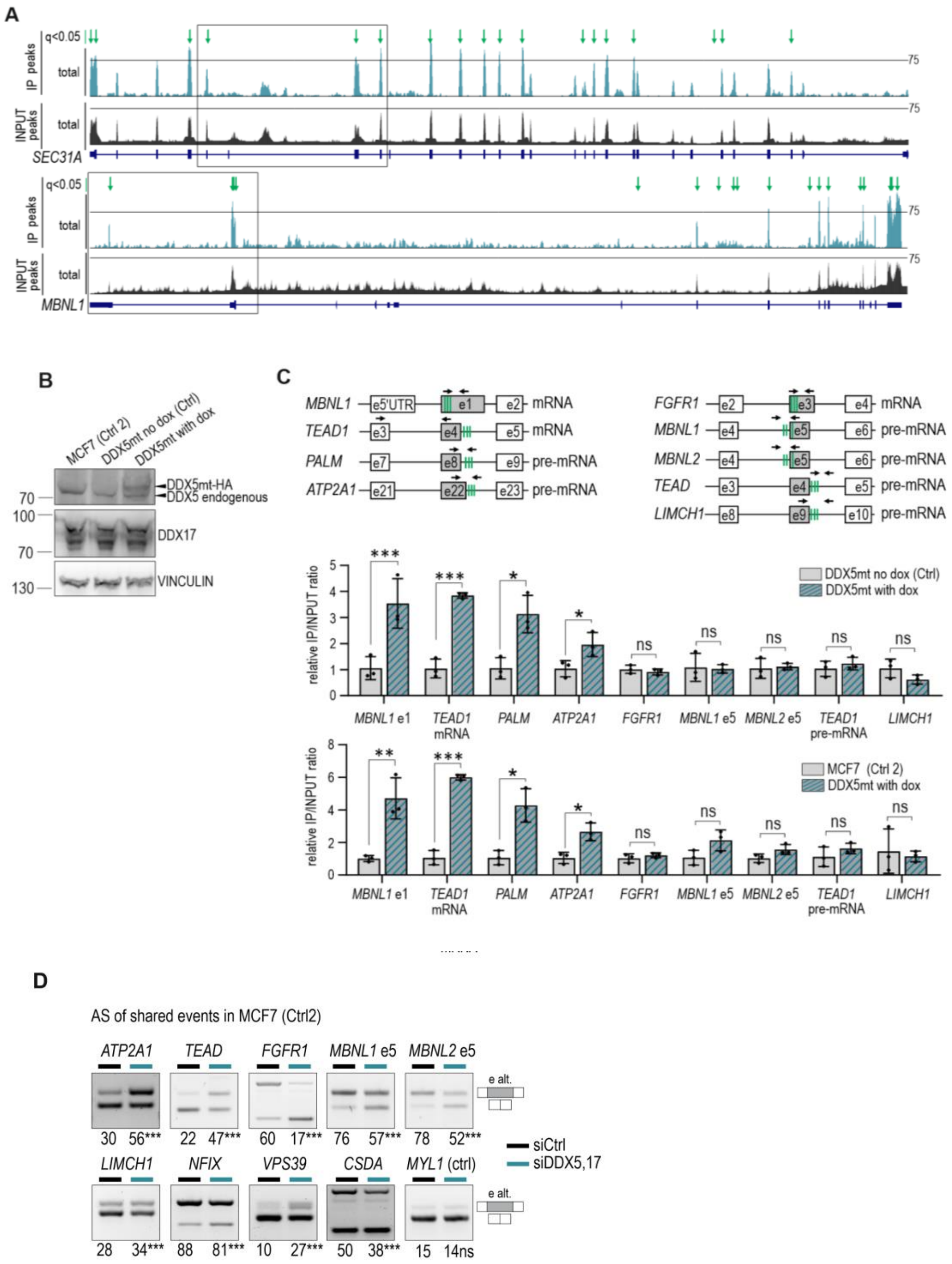


**Supplementary Figure S5**

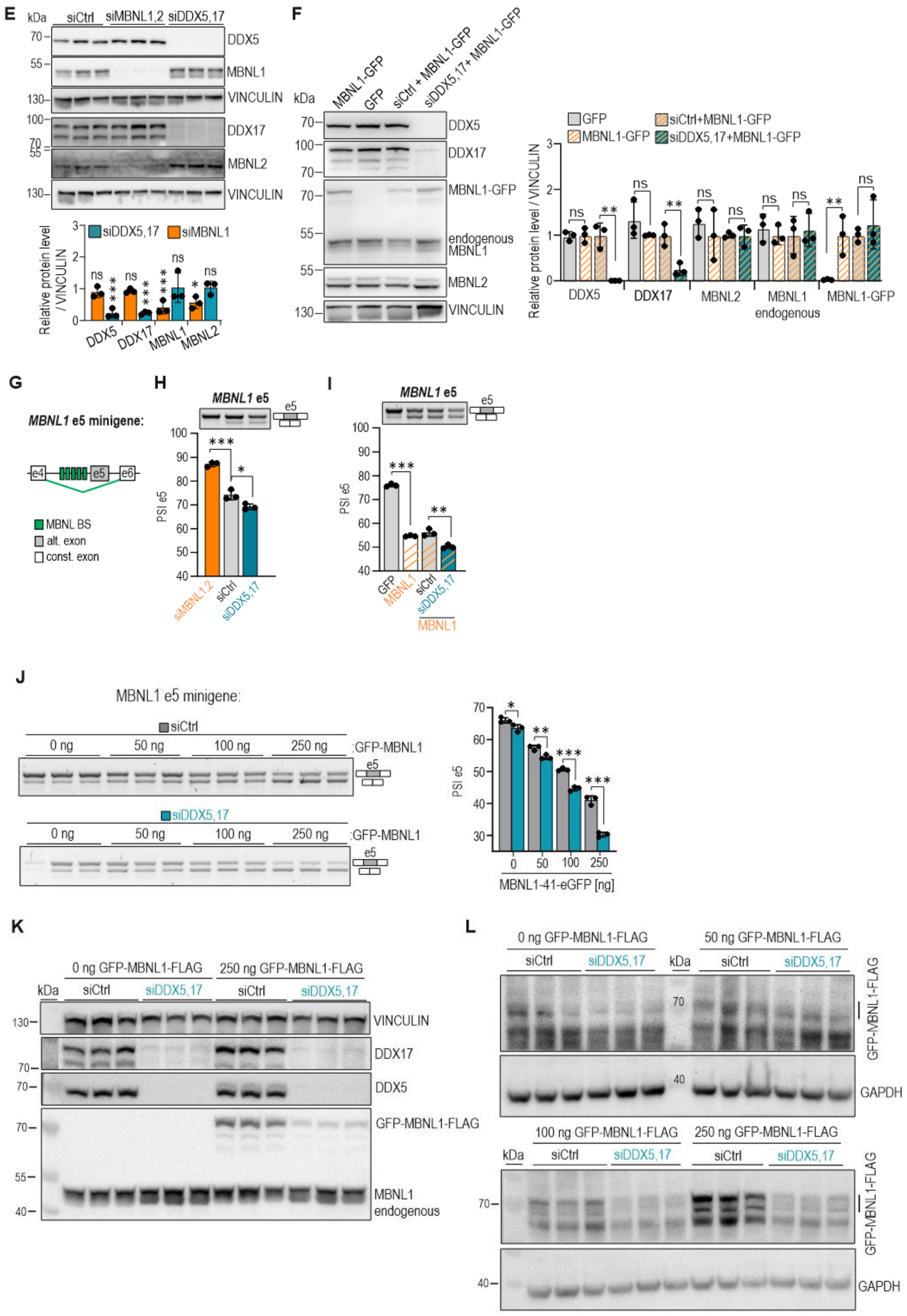


**Supplementary Figure S5**

**Supplementary Figure S5**

**DDX5,17 affect MBNL-dependent AS through most likely interfering in MBNL-RNA complexes.**

A) DDX5 RIP-seq for *SEC31A* and *MBNL1* transcripts. IP track (turquoise) and INPUT track (black) show reads coverage of DDX5 RIP-seq and input sequencing, respectively. Significant DDX5 enriched regions are marked with green arrows (q < 0.05 calculated by MACS as False Discovery Rate (FDR)). DDX5 RIP-seq tracks are presented with fixed vertical viewing range. The coverage by 75 reads is indicated by the black horizontal line. Regions of transcripts presented in the main Figure 4A are marked with a black box.

B) Western blot analyses of INPUT samples used in DDX5mt RIP. VINCULIN serves as a loading control. DDX5mt no dox constitutes the main control (Ctrl). MCF7 without genetic modifications constitutes the second control (Ctrl 2).

C) A scheme presenting fragments of analyzed transcripts in DDX5mt RIP, with indicated MBNL-dependent AS events marked with grey boxes, potential MBNL-binding sites with green boxes and location of primers used in qPCR marked with black arrows (*top*). qPCR analyses of selected transcripts in DDX5mt RIP (*bottom*). Data represent the mean PSI values +/- SD (n = 3). Statistical significance was calculated in reference to MCF7 DDX5mt cells without doxycycline induction using Student's t-test; ns, nonsignificant, ns, nonsignificant, ∗ for $P < 0.05$, ∗∗ for $P < 0.01$, ∗∗∗ for $P < 0.001$.

D) As in C) (*bottom*) but for MCF7 cell line without genetic modifications serving as a control (Ctrl 2).

E) Western blot analyses of DDX5, DDX17, MBNL1 and MBNL2 levels in HeLa cells upon knockdown of DDX5,17 or MBNL1,2 with siRNA. Immunoblot images of western blot with VINCULIN serving as a loading control (*left*). All samples derive from the same experiment and the blots were processed in parallel. Calculations of western blot (*right*). Data represent the mean values +/- SD (n = 3). Statistical significance was calculated in reference to control, siCtrl, using Student's t-test; ns, nonsignificant, ∗ for $P < 0.05$, ∗∗∗ for $P < 0.001$).

F) As in A) but with concomitant expression of MBNL1 on the background of siCtrl or siDDX5,17.

G) A scheme of the *MBNL1* e5 splicing minigene with multiple functional MBNL-binding sites upstream of the 3'ss marked with green. MBNL BS, MBNL-binding sites; alt exon, alternative exon; const. exon, constitutive exon.

H) Representative gels and calculations of RT-PCR analyses of the *MBNL1* e5 minigene splicing response upon GFP-MBNL1 or control GFP overexpression on the background of Mbnl1 and Mbnl2 double knock-out (Mbnl1,2 DKO) in mouse embryonic fibroblasts (MEFs). Data represent the mean PSI values +/- SD (n = 3). Statistical significance was calculated in reference to GFP using Student's t-test; ∗∗∗ for $P < 0.001$.

I) Representative gels and calculations of RT-PCR analyses of the *MBNL1* e5 minigenes' splicing response to transiently expressed GFP-MBNL1 in DDX5,17-depleted cells compared to control HeLa cells. Data represent the mean PSI values +/- SD (n = 3). Statistical significance was calculated in reference to siCtrl using Student's t-test; ∗∗∗ for $P < 0.001$.

J) Agarose gel representing RT-PCR analyses of MBNL1 minigene with alternative e5 at the background of DDX5,17 KD with simultaneous titration of GFP-MBNL1-FLAG expression vector at 50, 100 and 250 ng concentrations (*left*). Calculations of RT-PCR (*right*). Data represent the mean PSI values +/- SD (n = 3). Statistical significance was calculated in reference to control, siCtrl, using Student's t-test; ∗ for $P < 0.1$, ∗∗ for $P < 0.01$, ∗∗∗ for $P < 0.001$).

K) Western blot analyses of DDX5, DDX17, MBNL1 endogenous and GFP-MBNL1-FLAG in HeLa cells upon DDX5,17 KD and simultaneous titration of GFP-MBNL1-FLAG expression at 0 and 250 ng concentrations. VINCULIN serves as a loading control. Antibody against MBNL1 was used to visualize both endogenous and exogenous proteins.

L) Western blot analyses of GFP-MBNL1-FLAG in HeLa cells upon DDX5,17 KD and simultaneous titration of GFP-MBNL1-FLAG expression vector at 0, 50, 100 and 250 ng concentrations. GAPDH serves as a loading control. Antibody against FLAG was used to visualize the exogenous MBNL1.

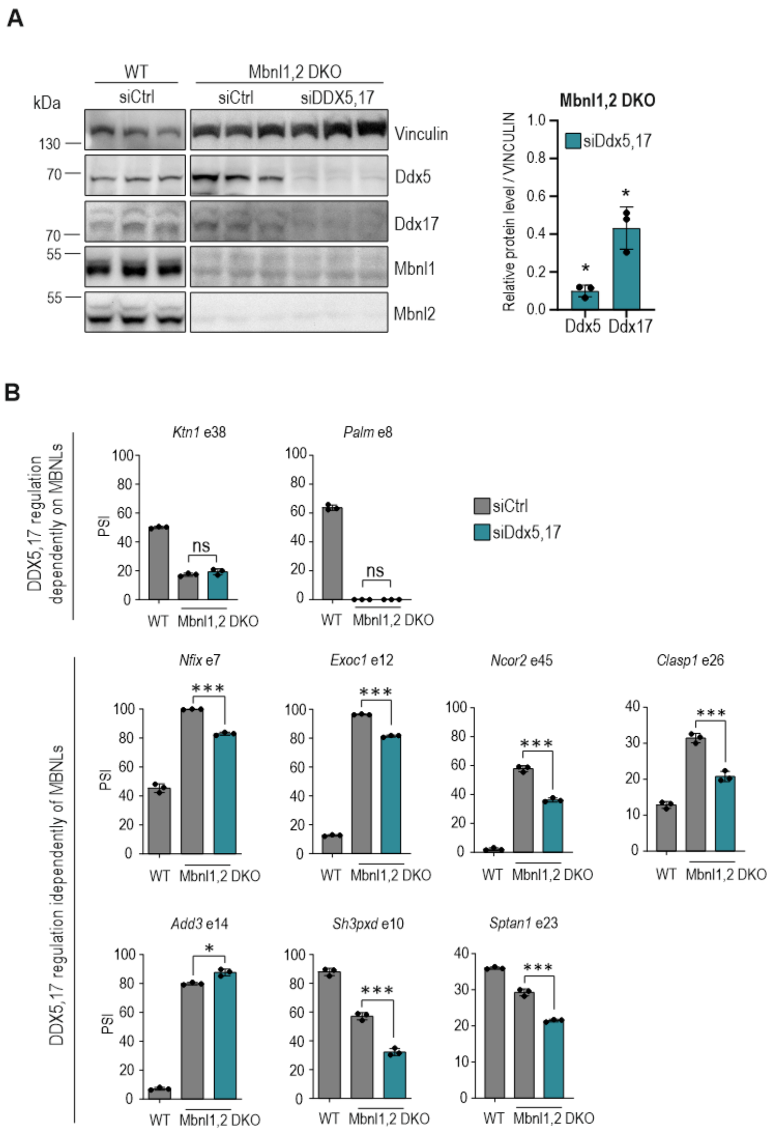


**Supplementary Figure S6**

**DDX5,17 modulate AS of certain shared events independent of MBNL1,2.**

A) Western blot analyses of Ddx5, Ddx17, Mbnl1 and Mbnl2 levels in MEF WT and MEF Mbnl1,2 DKO. Raw immunoblot images of western blot with Vinculin serving as a loading control (*left*). All samples derive from the same experiment and the blots were processed in parallel. Calculations of western blot (*right*). Data represent the mean PSI values +/- SD (n = 3). Statistical significance was calculated in reference to control (siCtrl) using Student's t-test; ∗ for $P < 0.05$).

B) RT-PCR splicing analyses of Mbnl-dependent AS events in MEFs upon Ddx5,17 silencing in the background of Mbnl1 and Mbnl2 double knock-out (DKO). Data represent the mean PSI values +/- SD (n = 3). Statistical significant was determined by Student's t-test; ns, nonsignificant, ∗ for P < 0.05, ∗∗∗ for P < 0.001.

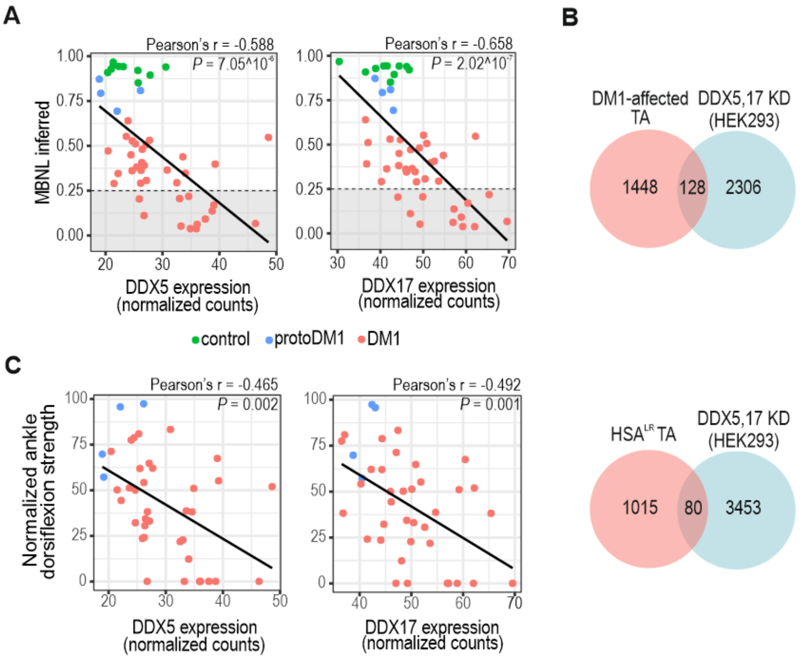


**Supplementary Figure S7**

**Expression levels of DDX5 and DDX17 in DM1-affected muscle tissues associate with the disease severity.**

A) Expression levels of *DDX5* and *DDX17* in 40 DM1-affected muscle tissues and 10 controls negatively correlate with the functional level of MBNL (MBNLinferred). Analyzed RNA-seq data were retrieved from an online available DMseq database [6]. MBNLinferred concentration was assesses based on PSI values of several MBNL-dependent AS events described elsewhere [7]. DESeq2-normalization method was used to calculate the expression level of the helicases. The grey area depicts the samples with MBNLinferred $< 0.25$ taken for AS analyses described in S7B. The strength of a linear association and its statistical significance was determined by Pearson's product-moment correlation.

B) Shared high-confidence AS events which splicing changed in DM1-affected muscle tissues (*top*) or $HSA^{LR}$ (*bottom*) and HEK293 DDX5,17 KD cells. Considered changes in splicing of simple cassette events have $|\Delta PSI| \geq 10$; $FDR \leq 0.05$. 12 DM1-affected samples used for this analyses are characterized by MBNL1inferred $<0.25$. Computed $\Delta PSI$ values from RNA-seq AS analyses were compared between HEK293 DDX5,17 KD cells (n = 3; [1]) and DM1-affected muscles (n = 40 DM1 or 12 controls [6]) or $HSA^{LR}$ (n = 4; [8]). Note that analyzed datasets substantially differ in a context of sample type and experimental procedure. $HSA^{LR}$, a mouse DM1-model expressing (CUG)exp RNA exclusively in skeletal muscles.

C) The increasing expression of the helicases negatively corelate with ankle dorsiflexion strength of DM1-afftected muscles. The latter data were retrieved from an online available DMseq database [6]. DESeq2-normalization method was used to calculate the expression level of the helicases. The strength of a linear association and its statistical significance was determined by Pearson's product-moment correlation.

## SUPLEMENTARY MATERIALS AND METHODS

### Splicing rescue assay

MCF7 stable cell lines expressing HA-tagged DDX5 wild type (DDX5wt) or DDX5 mutant (DDX5mt) were grown in high-glucose DMEM (Lonza) supplemented with 10% fetal bovine serum (FBS) (Sigma Aldrich) and 1x penicillin-streptomycin (Sigma Aldrich). All cells were grown at 37˚C and an atmosphere containing 5% $CO_2$. Genes were knocked down with siRNAs against MBNL1, MBNL2, DDX5 (3'UTR), DDX17 (3'UTR) or siCtrl [9, 10] (Sigma Aldrich) at 50 nM with RNAiMAX. After 24 hrs the cells were induced with 0.5 ug/ml of doxycycline followed by analysis of protein levels and AS after 48 hrs of incubation.

### C2C12 cell differentiation, RNA extraction and RT-PCR

All experiments in C2C12 cells (transfection and differentiation of the cells, RNA extraction and RT-PCR, protein extraction and western-blotting) were carried out as previously described [10]. Anti-Mbnl1 antibody was a gift from G.E. Morris.

### Protein subcellular fractionation

Approximately 5x10^6 HeLa cells per sample were treated with doxycycline for 120 h and subjected to fractionation. Nuclear and cytoplasmic protein fractions were isolated using an NE-PER™ Kit (Thermo Fisher) according to the manufacturer's protocol.

### *MBNL1* e5 minigene preparation and transfection

The *MBNL1* e5 minigene, analogous to what was previously described in [11], was prepared as follows. A region containing 51 nucleotides from the 3'-end of intron 3, exon 4, intron 4, exon 5, intron 5, exon 6, and 33 nucleotides of the 5'-end of intron 6 was amplified from HeLa genomic DNA using PCR primers carrying a 15-nt overhang complementary to the pcDNA3.1(-) plasmid. The PCR fragment was inserted into linearized pcDNA3.1(-) between the NotI and BamHI restriction sites. MBNL1 fragment amplification and linearization of pcDNA3.1(-) were carried out using Phusion HiFi DNA polymerase (Thermo Fisher) and CloneAmp HiFi PCR premix (Takara Bio), respectively. An In-Fusion HD Cloning kit (Takara Bio) was used for fast and directional cloning. The primer sets are listed in Supplementary Table S1. Cotransfection with 200 ng of the *MBNL1* e5 minigene and 250 ng of pEGFP-MBNL1-41 expression vector was preceded by siRNA treatment followed by 4 h of incubation. The cells were harvested 48 hr after transfection. Prior to transfection, the cells were plated in 12-well plates and transfected at 50-60% confluence with plasmids using Lipofectamine 3000 (Thermo Fisher) or with siRNA or antisense oligonucleotides using Lipofectamine RNAiMAX (Thermo Fisher) according to the manufacturer's protocol.

### GTEx-based analysis of gene expression and AS

Gene expression of *MBNL1*, *MBNL2*, *DDX5*, *DDX17* and AS analysis of *MBNL1* exon 7 in different human tissues were conducted based on data retrieved from the GTEx database from healthy individuals, including 802 skeletal muscle, 428 heart – atrial appendage, 240 brain - cerebellum and 577 lung tissues. To calculate the *MBNL1* exon 7 PSI, the junctional expression of chr3_152447774_152455541 (exon 6 - exon 7) and chr3_152447774_152456266 (exon 6 - exon 8) was determined.

### RNA-seq data analyses

Publicly available RNA-seq data were retrieved from public repositories including DM1-affected muscles and control [6] and HSALR and WT mice (PRJNA625451 [8]). Reads were aligned to the human hg38 or mouse mm10 genomes. Salmon [12] was used for transcript expression quantification and differential gene expression analysis was performed using DESeq2 [13]. For AS analysis, reads were aligned using STAR [14] followed by rMATS [15] (version 4) analysis. Results of AS analyses and expression analyses and are listed in Supplementary Table S2.

**Supplementary Table S1 (Excell).** List of primers, siRNAs and SSOs [2, 3, 7-10, 16-24]

**Supplementary Table S2 (Excell).** Alternative splicing and gene expression analyses based on data retrieved from publicly available RNA-seq and Affymetrix exon array experiments.

**REFERENCES**


(1) Ben Ameur, L.; Marie, P.; Thenoz, M.; Giraud, G.; Combe, E.; Claude, J. B.; Lemaire, S.; Fontrodona, N.; Polveche, H.; Bastien, M.; et al. Intragenic recruitment of NF-kappa B drives splicing modifications upon activation by the oncogene Tax of HTLV-1. *Nat. Commun.* **2020**, *11* (1), 12, Article. DOI: 10.1038/s41467-020-16853-x.
(2) Han, H.; Irimia, M.; Ross, P. J.; Sung, H. K.; Alipanahi, B.; David, L.; Golipour, A.; Gabut, M.; Michael, I. P.; Nachman, E. N.; et al. MBNL proteins repress ES-cell-specific alternative splicing and reprogramming. *Nature* **2013**, *498* (7453), 241-248, Article. DOI: 10.1038/nature12270.
(3) Ray, D. MBNL1 regulated alternative splicing of MAP2K7 promotes de-differentiation of cancer cells by activating JNK. *Cancer Research* **2020**, *80* (16), 1, Meeting Abstract. DOI: 10.1158/1538-7445.am2020-3778.
(4) Brinegar, A. E.; Xia, Z.; Loehr, J. A.; Li, W.; Rodney, G. G.; Cooper, T. A. Extensive alternative splicing transitions during postnatal skeletal muscle development are required for calcium handling functions. *eLife* **2017**, *6*, 21, Article. DOI: 10.7554/eLife.27192.
(5) Weyn-Vanhentenryck, S. M.; Feng, H. J.; Ustianenko, D.; Duffie, R.; Yan, Q. H.; Jacko, M.; Martinez, J. C.; Goodwin, M.; Zhang, X. G.; Hengst, U.; et al. Precise temporal regulation of alternative splicing during neural development. *Nat. Commun.* **2018**, *9*, 17, Article. DOI: 10.1038/s41467-018-04559-0.
(6) Wang, E. T.; Treacy, D.; Eichinger, K.; Struck, A.; Estabrook, J.; Olafson, H.; Wang, T. T.; Bhatt, K.; Westbrook, T.; Sedehizadeh, S.; et al. Transcriptome alterations in myotonic dystrophy skeletal muscle and heart. *Hum. Mol. Genet.* **2019**, *28* (8), 1312-1321. DOI: 10.1093/hmg/ddy432.
(7) Wagner, S. D.; Struck, A. J.; Gupta, R.; Farnsworth, D. R.; Mahady, A. E.; Eichinger, K.; Thornton, C. A.; Wang, E. T.; Berglund, J. A. Dose-Dependent Regulation of Alternative Splicing by MBNL Proteins Reveals Biomarkers for Myotonic Dystrophy. *PLoS Genet.* **2016**, *12* (9), 24, Article. DOI: 10.1371/journal.pgen.1006316.
(8) Tanner, M. K.; Tang, Z. Z.; Thornton, C. A. Targeted splice sequencing reveals RNA toxicity and therapeutic response in myotonic dystrophy. *Nucleic Acids Res.* **2021**, *49* (4), 2240-2254, Article. DOI: 10.1093/nar/gkab022.
(9) Sznajder, L. J.; Michalak, M.; Taylor, K.; Cywoniuk, P.; Kabza, M.; Wojtkowiak-Szlachcic, A.; Matloka, M.; Konieczny, P.; Sobczak, K. Mechanistic determinants of MBNL activity. *Nucleic Acids Research* **2016**, *44* (21), 10326-10342, Article. DOI: 10.1093/nar/gkw915.
(10) Dardenne, E.; Espinoza, M. P.; Fattet, L.; Germann, S.; Lambert, M. P.; Neil, H.; Zonta, E.; Mortada, H.; Gratadou, L.; Deygas, M.; et al. RNA Helicases DDX5 and DDX17 Dynamically Orchestrate Transcription, miRNA, and Splicing Programs in Cell Differentiation. *Cell Reports* **2014**, *7* (6), 1900-1913. DOI: 10.1016/j.celrep.2014.05.010.
(11) Gates, D. P.; Coonrod, L. A.; Berglund, J. A. Autoregulated Splicing of muscleblind-like 1 (MBNL1) Pre-mRNA. *Journal of Biological Chemistry* **2011**, *286* (39), 34224-34233. DOI: 10.1074/jbc.M111.236547.
(12) Patro, R.; Duggal, G.; Love, M. I.; Irizarry, R. A.; Kingsford, C. Salmon provides fast and bias-aware quantification of transcript expression. *Nat. Methods* **2017**, *14* (4), 417-+, Article. DOI: 10.1038/nmeth.4197.

(13) Love, M. I.; Huber, W.; Anders, S. Moderated estimation of fold change and dispersion for RNA-seq data with DESeq2. *Genome Biol.* **2014**, *15* (12), 38, Article. DOI: 10.1186/s13059-014-0550-8.
(14) Dobin, A.; Davis, C. A.; Schlesinger, F.; Drenkow, J.; Zaleski, C.; Jha, S.; Batut, P.; Chaisson, M.; Gingeras, T. R. STAR: ultrafast universal RNA-seq aligner. *Bioinformatics* **2013**, *29* (1), 15-21, Article. DOI: 10.1093/bioinformatics/bts635.
(15) Shen, S. H.; Park, J. W.; Lu, Z. X.; Lin, L.; Henry, M. D.; Wu, Y. N.; Zhou, Q.; Xing, Y. rMATS: Robust and flexible detection of differential alternative splicing from replicate RNA-Seq data. *Proc. Natl. Acad. Sci. U. S. A.* **2014**, *111* (51), E5593-E5601, Article. DOI: 10.1073/pnas.1419161111.
(16) Dixon, D. M.; Choi, J.; El-Ghazali, A.; Park, S. Y.; Roos, K. P.; Jordan, M. C.; Fishbein, M. C.; Comai, L.; Reddy, S. Loss of muscleblind-like 1 results in cardiac pathology and persistence of embryonic splice isoforms. *Scientific Reports* **2015**, *5*, 13, Article. DOI: 10.1038/srep09042.
(17) Wang, E. T.; Ward, A. J.; Cherone, J. M.; Giudice, J.; Wang, T. T.; Treacy, D. J.; Lambert, N. J.; Freese, P.; Saxena, T.; Cooper, T. A.; et al. Antagonistic regulation of mRNA expression and splicing by CELF and MBNL proteins. *Genome Research* **2015**, *25* (6), 858-871, Article. DOI: 10.1101/gr.184390.114.
(18) Fugier, C.; Klein, A. F.; Hammer, C.; Vassilopoulos, S.; Ivarsson, Y.; Toussaint, A.; Tosch, V.; Vignaud, A.; Ferry, A.; Messaddeq, N.; et al. Misregulated alternative splicing of BIN1 is associated with T tubule alterations and muscle weakness in myotonic dystrophy. *Nature Medicine* **2011**, *17* (6), 720-U112, Article. DOI: 10.1038/nm.2374.
(19) Mersaoui, S. Y.; Yu, Z. B.; Coulombe, Y.; Karam, M.; Busatto, F. F.; Masson, J. Y.; Richard, S. Arginine methylation of the DDX5 helicase RGG/RG motif by PRMT5 regulates resolution of RNA:DNA hybrids. *Embo Journal* **2019**, *38* (15), 20, Article. DOI: 10.15252/embj.2018100986.
(20) Sithole, N.; Williams, C. A.; Vaughan, A. M.; Kenyon, J. C.; Lever, A. M. L. DDX17 Specifically, and Independently of DDX5, Controls Use of the HIV A4/5 Splice Acceptor Cluster and Is Essential for Efficient Replication of HIV. *Journal of Molecular Biology* **2018**, *430* (18), 3111-3128, Article. DOI: 10.1016/j.jmb.2018.06.052.
(21) Sarbassov, D. D.; Guertin, D. A.; Ali, S. M.; Sabatini, D. M. Phosphorylation and regulation of Akt/PKB by the rictor-mTOR complex. *Science* **2005**, *307* (5712), 1098-1101, Article. DOI: 10.1126/science.1106148.
(22) Holt, I.; Jacquemin, V.; Fardaei, M.; Sewry, C. A.; Butler-Browne, G. S.; Furling, D.; Brook, J. D.; Morris, G. E. Muscleblind-Like Proteins Similarities and Differences in Normal and Myotonic Dystrophy Muscle. *American Journal of Pathology* **2009**, *174* (1), 216-227, Article. DOI: 10.2353/ajpath.2009.080520.
(23) Tabaglio, T.; Low, D. H. P.; Teo, W. K. L.; Goy, P. A.; Cywoniuk, P.; Wollmann, H.; Ho, J.; Tan, D.; Aw, J.; Pavesi, A.; et al. MBNL1 alternative splicing isoforms play opposing roles in cancer. *Life Science Alliance* **2018**, *1* (5). DOI: 10.26508/lsa.201800157.
(24) Wang, E. T.; Cody, N. A. L.; Jog, S.; Biancolella, M.; Wang, T. T.; Treacy, D. J.; Luo, S. J.; Schroth, G. P.; Housman, D. E.; Reddy, S.; et al. Transcriptome-wide Regulation of Pre-mRNA Splicing and mRNA Localization by Muscleblind Proteins. *Cell* **2012**, *150* (4), 710-724, Article. DOI: 10.1016/j.cell.2012.06.041.
